# Supplementary material for: Sirt2 facilitates hepatic glucose uptake by deacetylating glucokinase regulatory protein
Source: Nat Commun. 2018 Jan 2;9:30. doi: 10.1038/s41467-017-02537-6 (PMC5750207; doi:10.1038/s41467-017-02537-6)
Supplement: Supplementary file 1 — Supplementary Information [file 41467_2017_2537_MOESM1_ESM.pdf]

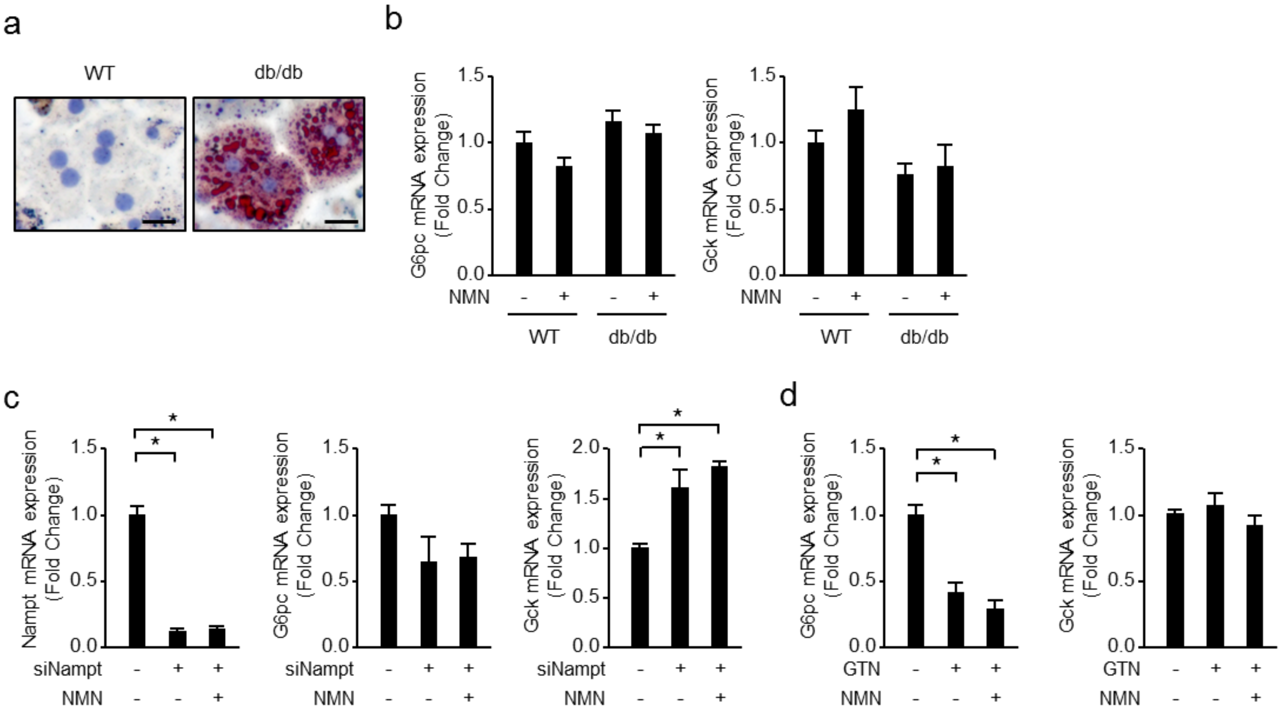

Supplementary Figure 1. NAD+ and hepatic expression of Gck and G6pc. (**a**) Oil red O staining in primary hepatocytes derived from lean and db/db mice. Scale bars indicate 20 μm. (**b**) Expressions of G6pc and Gck mRNA in primary hepatocytes derived from lean and db/db mice (n = 3). (**c**) Effect of siRNA-mediated knockdown of Nampt and nicotinamide mononucleotide (NMN) on mRNA expressions of G6pc, Gck, and Nampt in mouse primary hepatocytes (n = 3). (**d**) Effect of gallotannin (GTN) and NMN on mRNA expressions of G6pc and Gck in mouse primary hepatocytes (n = 3). *$P < 0.05$; one-way analysis of variance (ANOVA) with the Fisher's PLSD *post hoc* test (**b–d**). Error bars show the standard error of the mean (s.e.m).

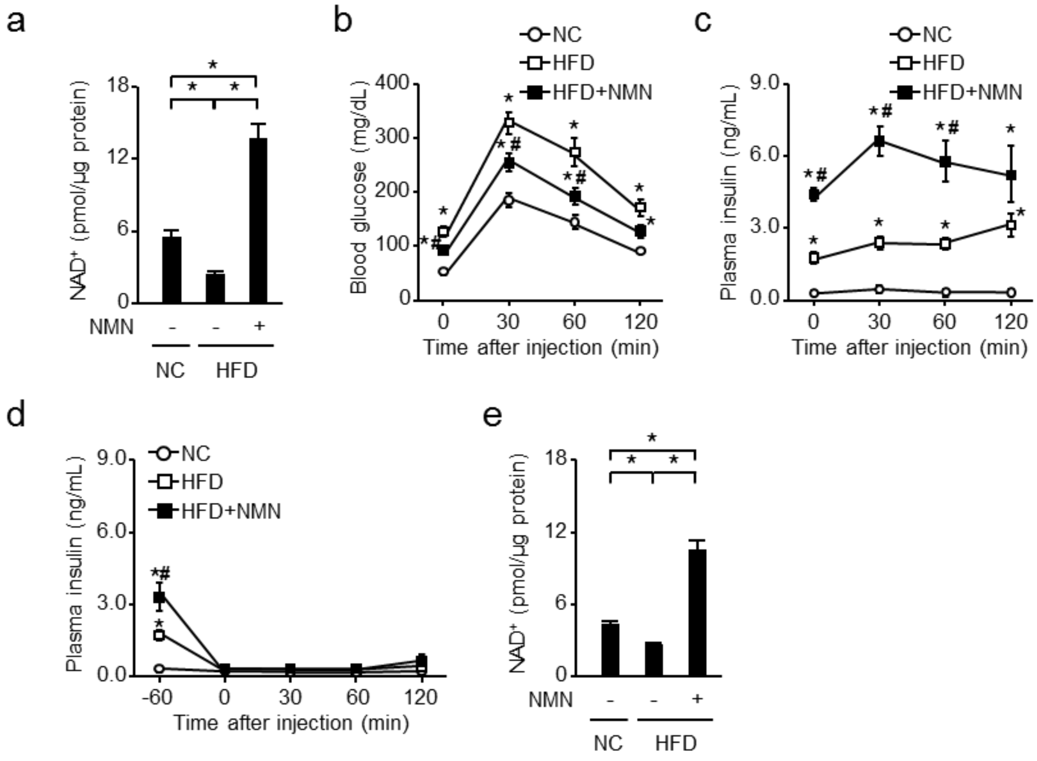

Supplementary Figure 2. Glucose tolerance testing with and without NMN administration. (**a**–**c**) NAD+ level (*n* = 3) (**a**) in the liver and blood glucose (*n* = 5) (**b**) and plasma insulin (**c**) (*n* = 5) levels in mice fed a normal chow diet (NC), high-fat diet (HFD), and a HFD and treated intraperitoneally with NMN after glucose loading (1 g/kg). (**d**,**e**) Plasma insulin level (*n* = 5) (**d**) and NAD+ level in the liver (**e**) (*n* = 3) in NC, HFD, and HFD mice treated with NMN after glucose administration (1 g/kg) with concomitant continuous dosing of somatostatin (3 µg/kg/min). *P < 0.05; one-way ANOVA with the Fisher's PLSD *post hoc* test (**a**,**e**), *P < 0.05, versus NC; #P < 0.05, HFD versus HFD with NMN in **b**–**d**; one-way ANOVA with the Fisher's PLSD *post hoc* test. Error bars show s.e.m.

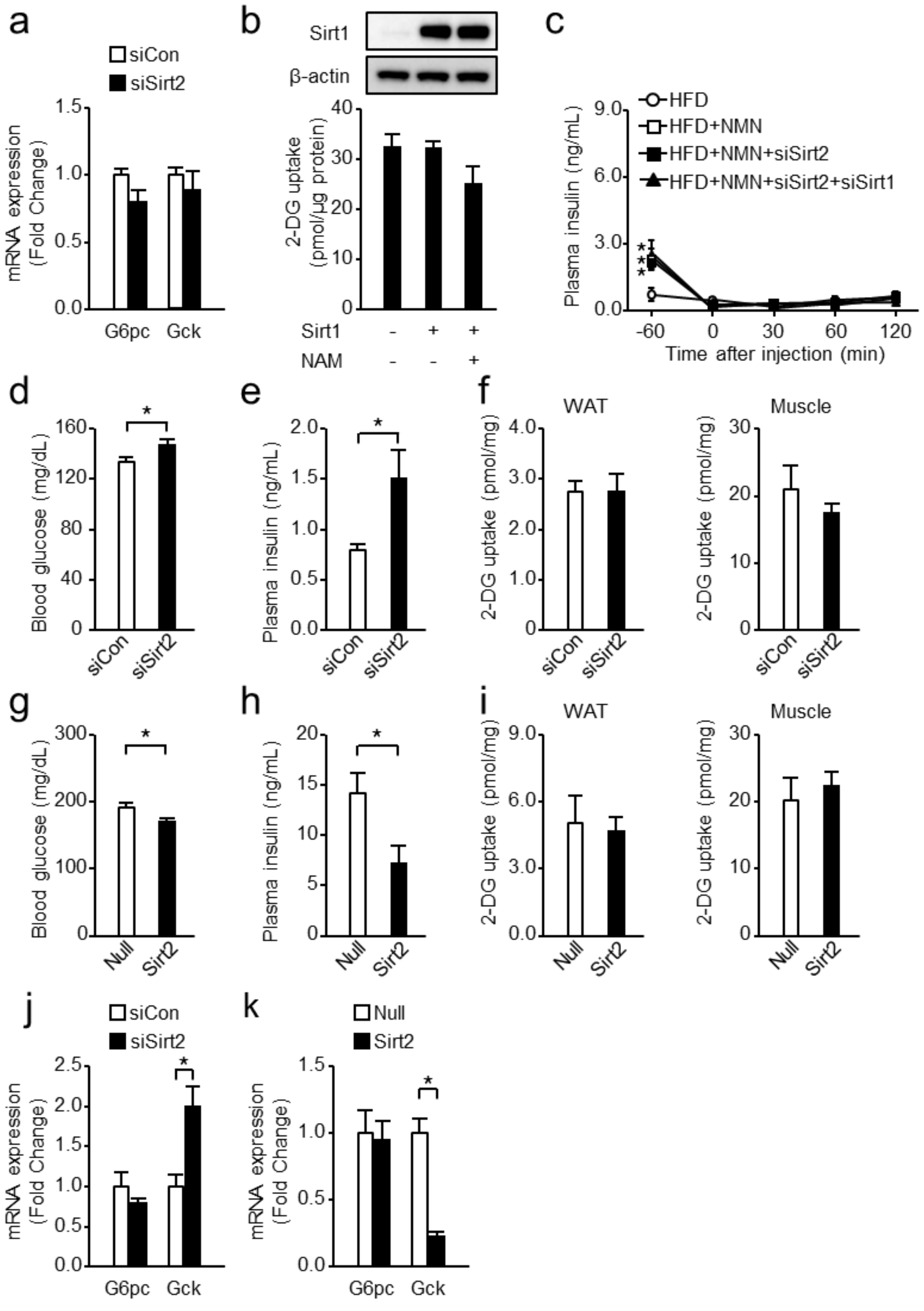

a
siCon
siSirt2
mRNA expression (Fold Change)
G6pc
Gck
b
Sirt1
β-actin
2-DG uptake (pmol/μg protein)
Sirt1 - + +
NAM - - +
c
HFD
HFD+NMN
HFD+NMN+siSirt2
HFD+NMN+siSirt2+siSirt1
Plasma insulin (ng/mL)
Time after injection (min)
d
Blood glucose (mg/dL)
siCon
siSirt2
e
Plasma insulin (ng/mL)
f
WAT
Muscle
2-DG uptake (pmol/mg)
g
Blood glucose (mg/dL)
Null
Sirt2
h
Plasma insulin (ng/mL)
i
WAT
Muscle
2-DG uptake (pmol/mg)
j
siCon
siSirt2
mRNA expression (Fold Change)
k
Null
Sirt2
mRNA expression (Fold Change)

Supplementary Figure 3. Phenotypes of mice with hepatic Sirt2 knockdown or overexpression. (**a**) Gene expression of G6pc and Gck in mouse primary hepatocytes treated with or without siSirt2 (n = 3). (**b**) Effect of adenovirus-mediated overexpression of Sirt1 on 2-DG uptake in primary hepatocytes derived from db/db mouse in the presence or absence of NAM (n = 4). (**c**) Plasma insulin level during glucose tolerance testing (1 g/kg) in HFD mice treated with NMN in the presence or absence of hepatic Sirt2 knockdown or Sirt2 and Sirt1 double knockdown during continuous dosing of somatostatin (n = 5). (**d**,**e**) Blood glucose (**d**) and plasma insulin (**e**) levels of hepatic Sirt2 knockdown lean mice under free-feeding conditions (n = 9). (**f**) Uptake of 2-deoxyglucose in the white adipose tissue (WAT) and skeletal muscle of hepatic Sirt2 knockdown lean mice after glucose administration (2 g/kg) (n = 4). (**g**,**h**) Levels of blood glucose (**g**) and plasma insulin (**h**) of HFD-fed mice with adenovirus-mediated hepatic Sirt2 overexpression under free-feeding conditions (n = 7). (**i**) Uptake of 2-deoxyglucose in the WAT and skeletal muscle in hepatic Sirt2-overexpressing lean mice after glucose administration (1 g/kg) (n = 5). (**j**,**k**) Hepatic mRNA expression of G6pc and Gck in hepatic Sirt2 knockdown mice (n = 4) (**j**) and in HFD-fed mice with adenovirus-mediated hepatic Sirt2 overexpression (n = 5) (**k**). *$P < 0.05$; Student's t-test (**a**,**d**–**k**) and one-way ANOVA with the Fisher's PLSD *post hoc* test (**b**). *$P < 0.05$, versus HFD in **c**; one-way ANOVA with the Fisher's PLSD *post hoc* test. Error bars show s.e.m.

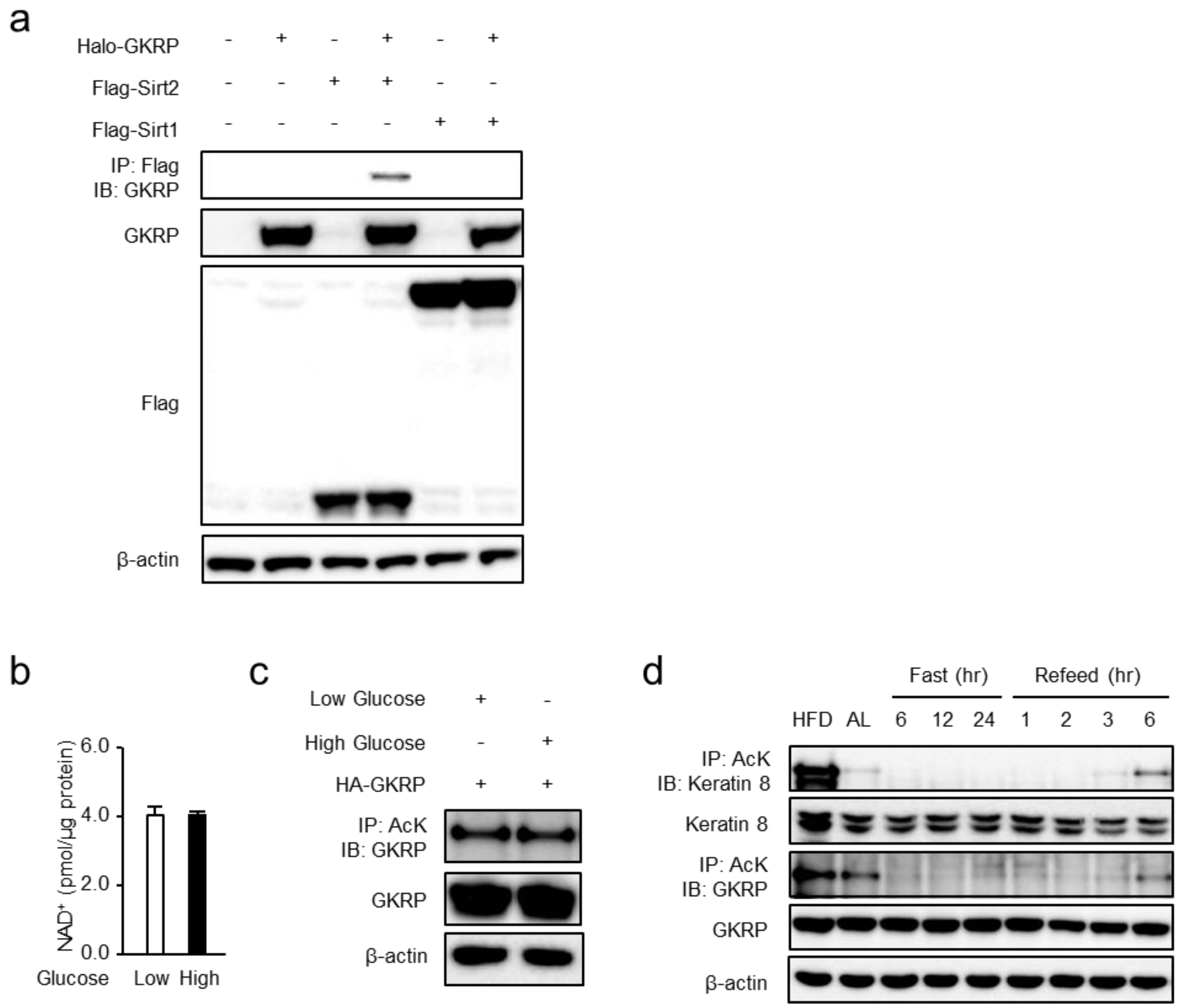

Supplementary Figure 4. Acetylated GKRP levels in hepatocytes cultured under low- or high-glucose conditions and in the liver under fasted/refed conditions. (**a**) Immunoprecipitation and immunoblot analysis of the interaction between Sirt2 and GKRP or Sirt1 and GKRP in HEK293 cells expressing Flag-tagged Sirt2, Flag-tagged Sirt1, or Halo-tagged GKRP. (**b**) NAD+ level in mouse primary hepatocytes cultured in low- or high-glucose medium (*n* = 5). (**c**) Acetylation level of GKRP in mouse primary hepatocytes cultured in low- or high-glucose medium. (**d**) Successive comparison of lysine acetylation levels of keratin 8 and GKRP in the liver of NC-fed mice under a fasted/refed condition for the indicated times, and under ad libitum-fed conditions (AL). Error bars show s.e.m.

Supplementary Figure 5

a

K43: K*ADAEKIVQ

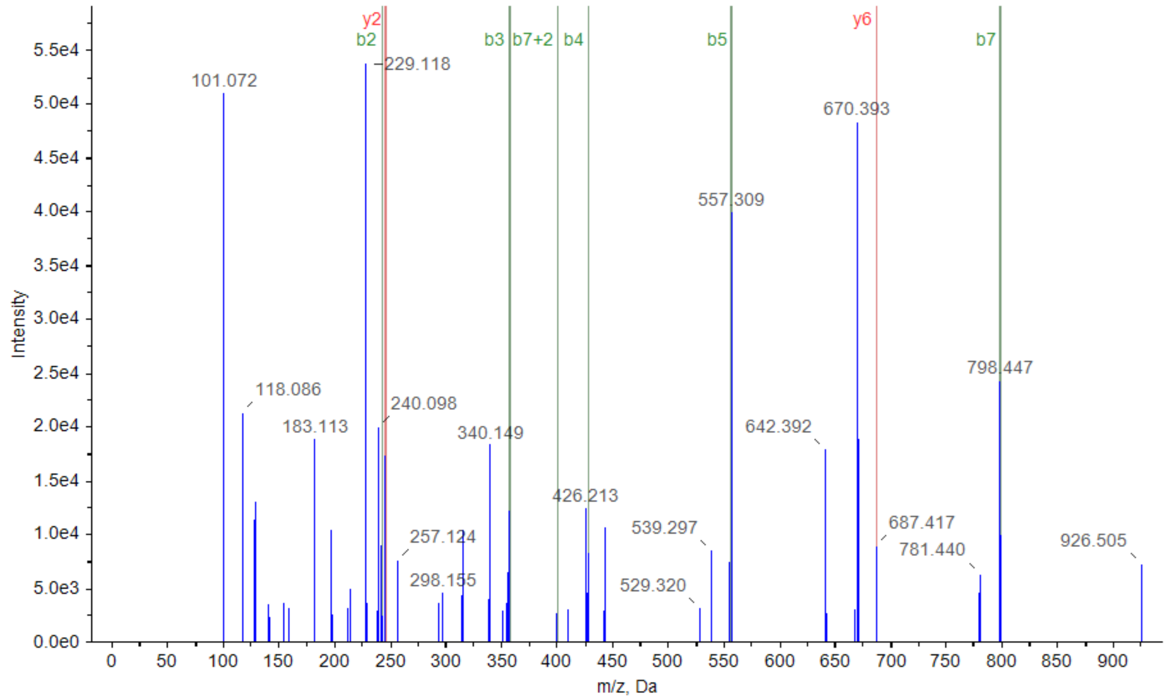

b

K126: NQLMK*GLGQK

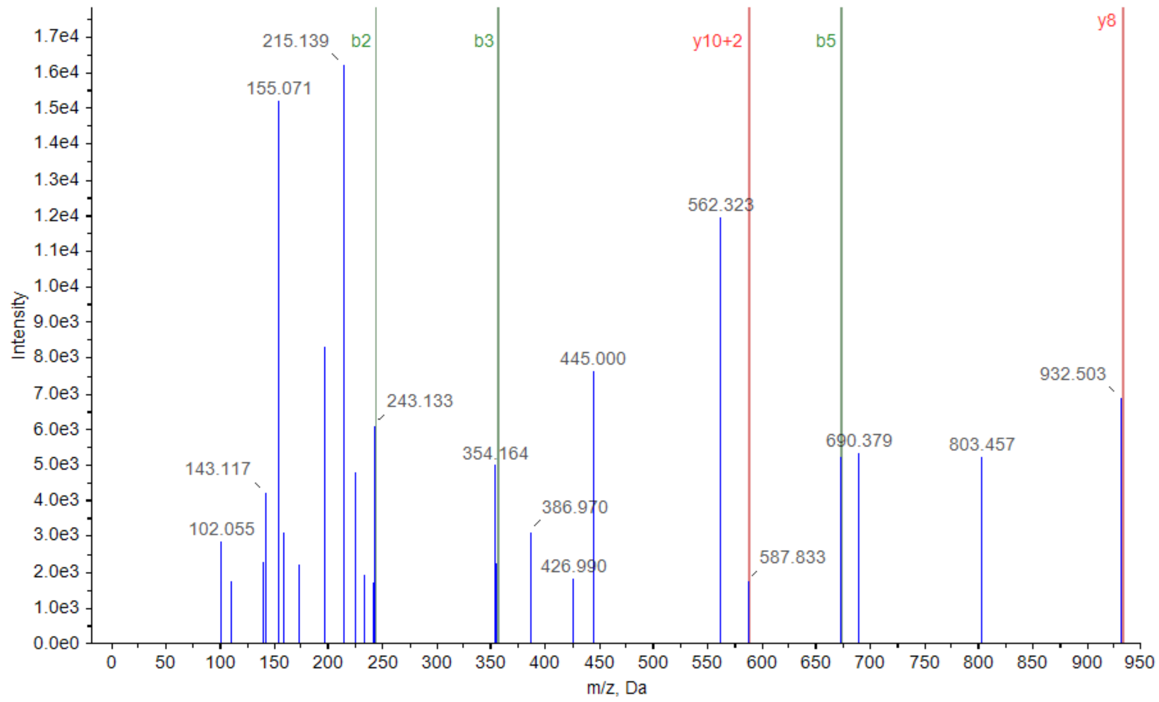

Supplementary Figure 5

c

K165: GIEELKK*VAAG

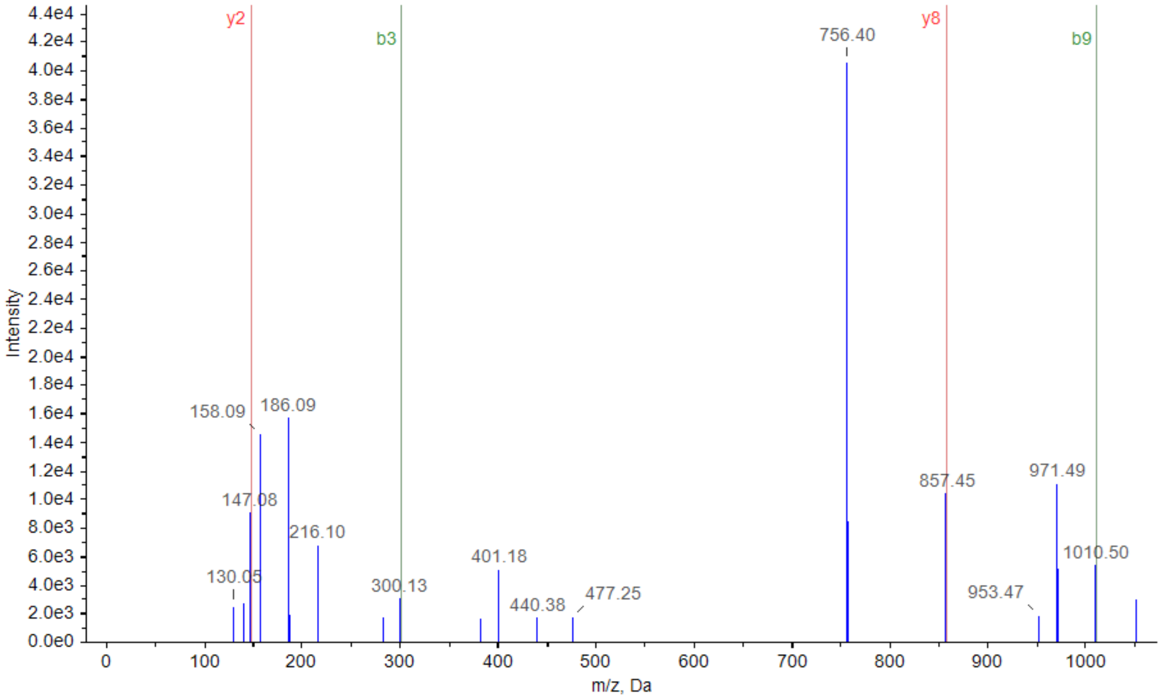

d

K235: QK*MQEKQ

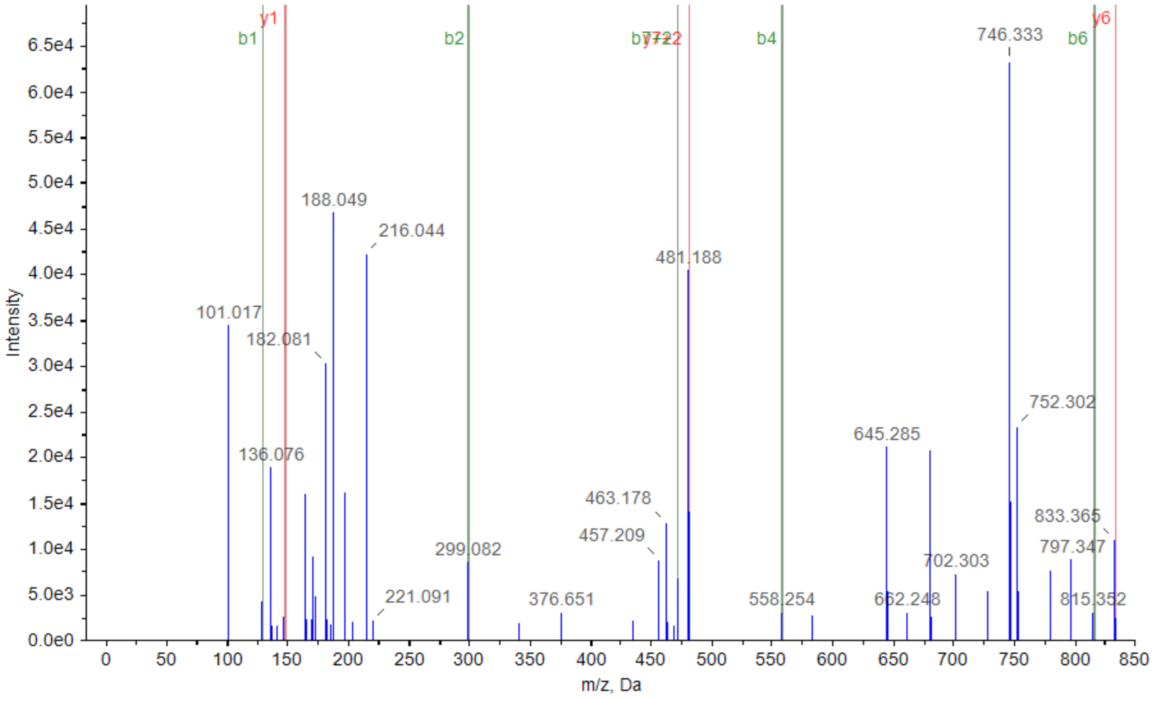

Supplementary Figure 5

e

K267: GSATK*ILLE

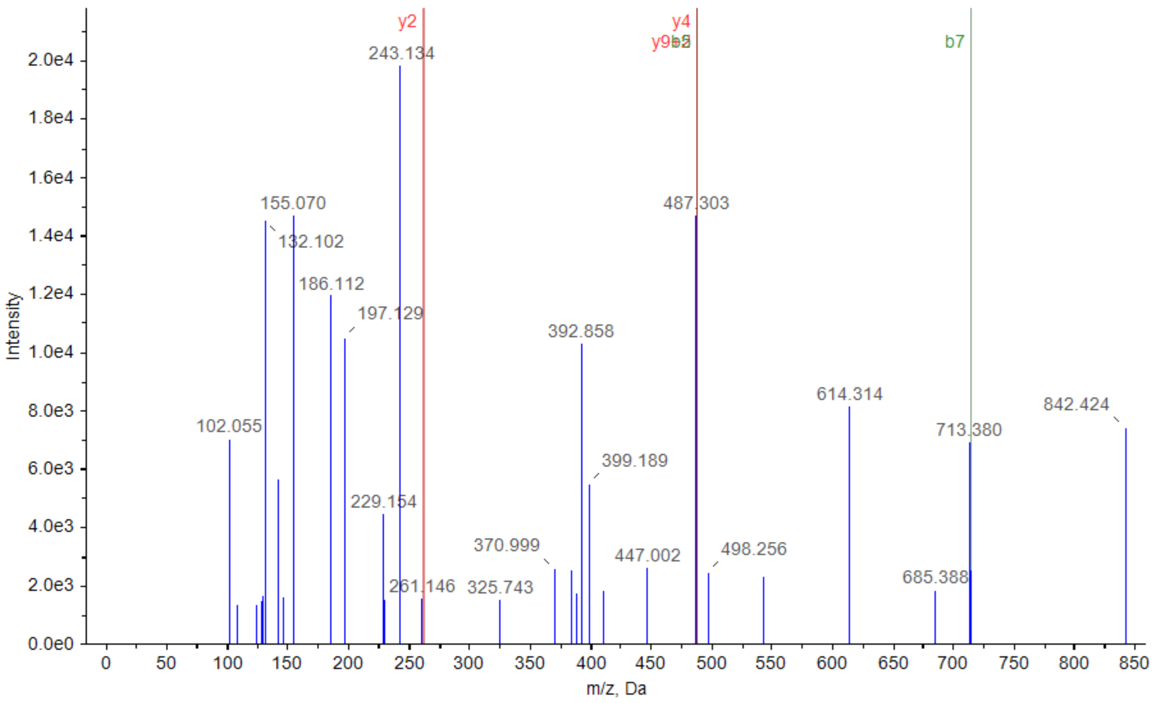

f

K279: LAAHK*TVDQGVV

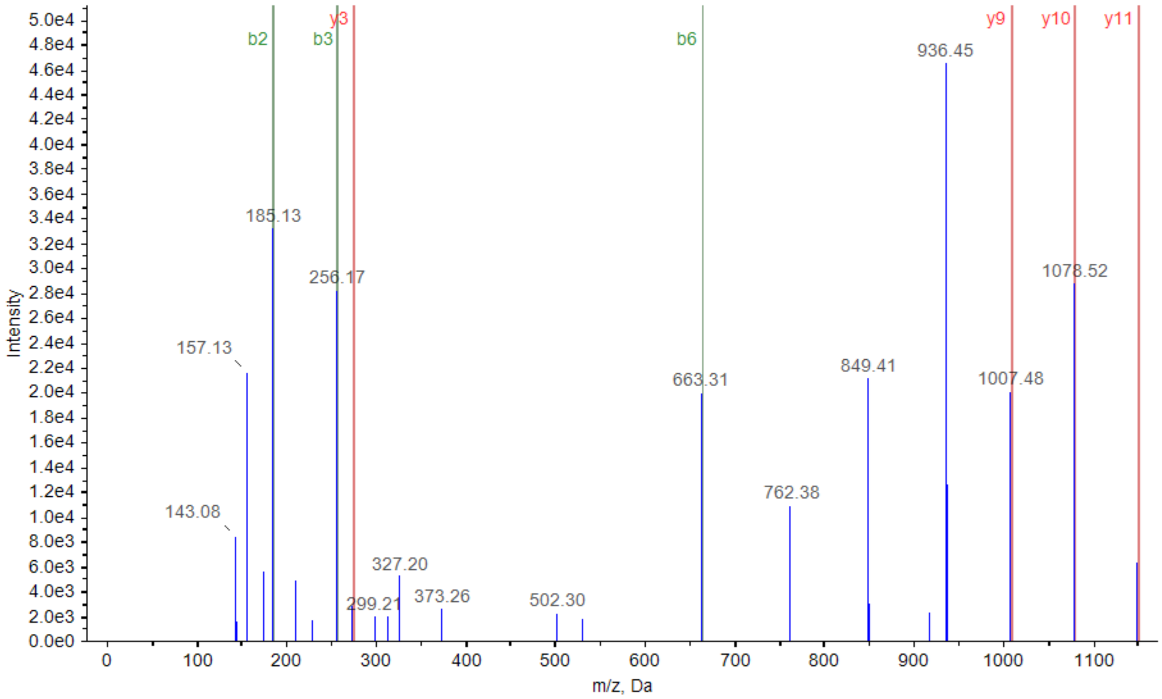

g

K312: K*IATLTKQV

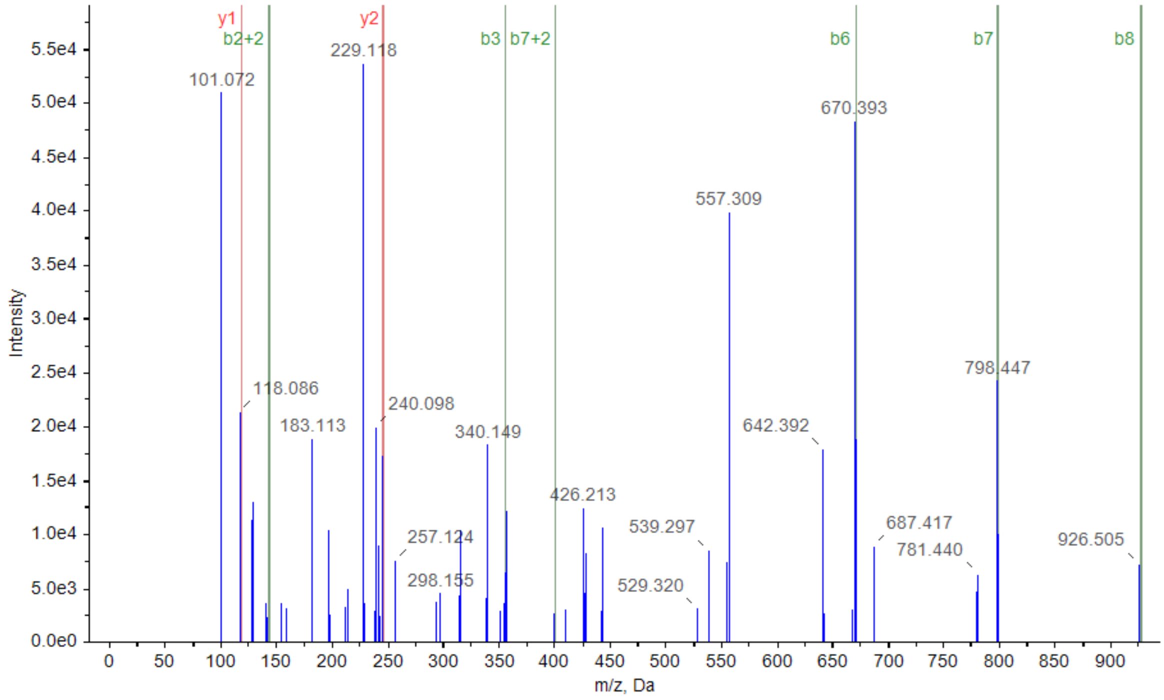

h

K327: ISLEKK*

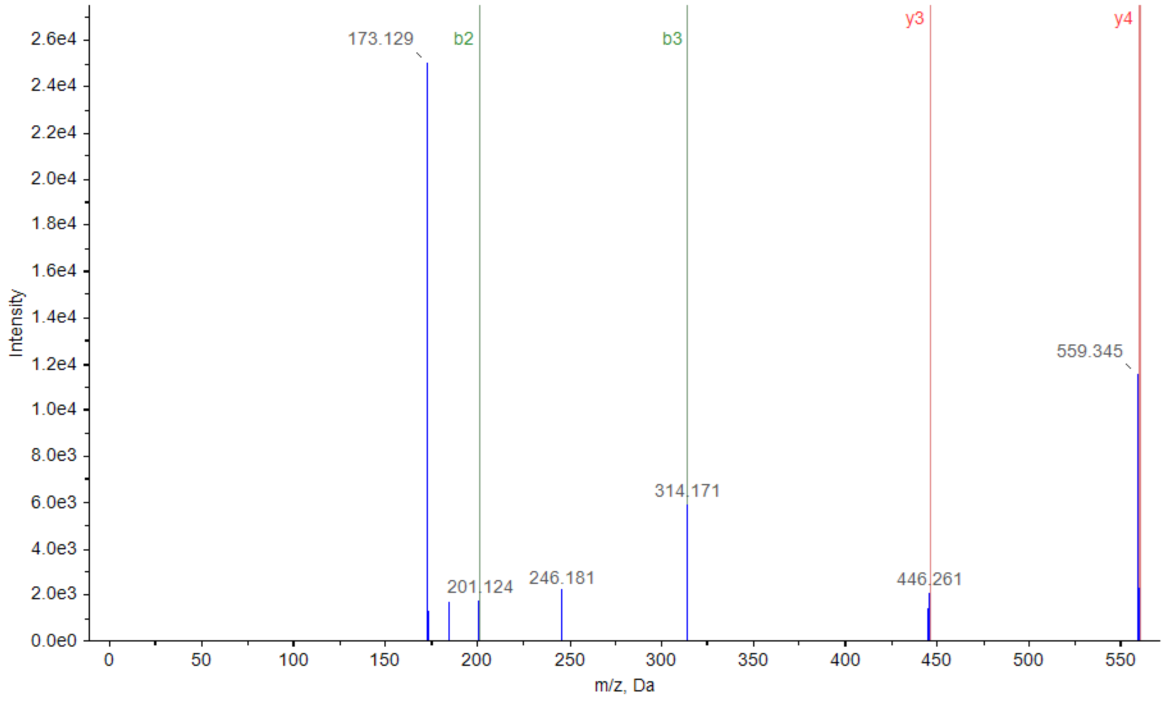

Supplementary Figure 5. Mass spectrometry analysis to determine the acetylation site of GKRP. (**a–h**) Detection of acetylated lysines 43, 126, 235, 267, 279, 312, and 327 in mouse GKRP by MS/MS analysis. MS/MS spectra of the GKRP-derived peptides are shown, with the C-terminal y ions and N-terminal b ions labelled as assigned. K*, acetylated lysine.

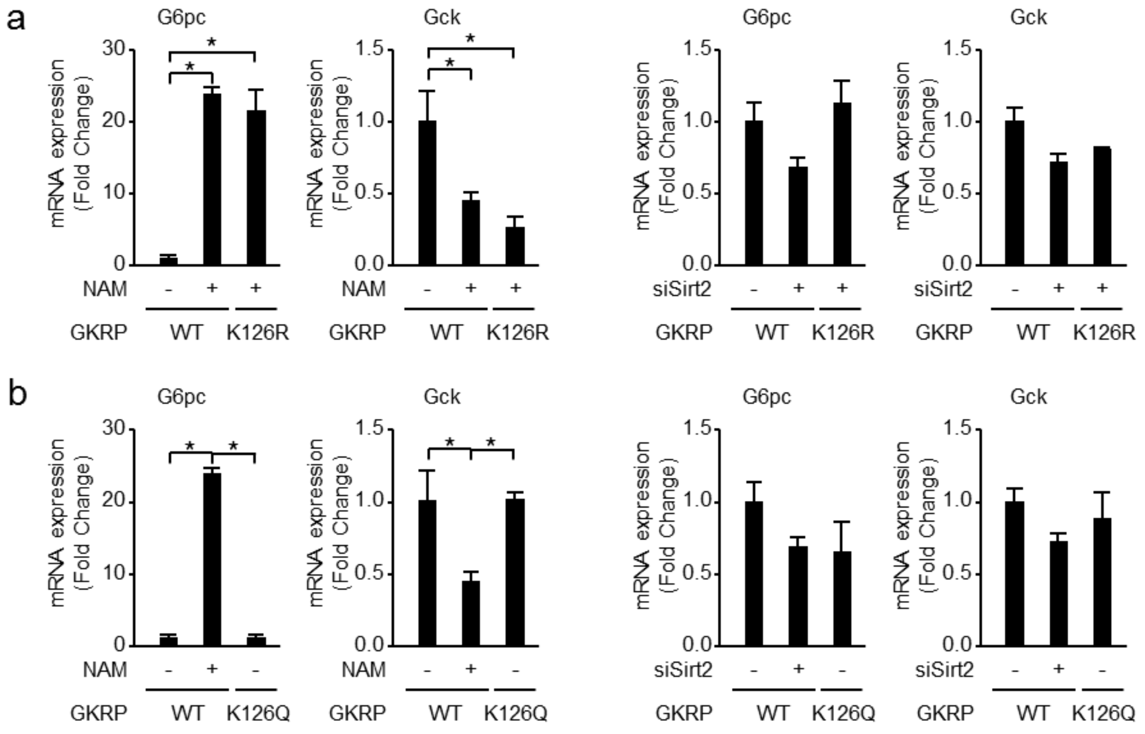

Supplementary Figure 6. GKRP-K126R or K126Q mutant has no effect on *Gck* and *G6pc* mRNA expression in primary hepatocytes. (**a**) Gene expression of *G6pc* and *Gck* in mouse primary hepatocytes transfected with adenovirus encoding Flag-tagged GKRP-wild-type (GKRP-WT) and Flag-tagged GKRP-K126R with NAM treatment or Sirt2 knockdown ($n = 4$). (**b**) Gene expression of *G6pc* and *Gck* in mouse primary hepatocytes transfected with adenovirus encoding Flag-tagged GKRP-WT and Flag-tagged GKRP-K126Q with NAM treatment or Sirt2 knockdown ($n = 4$). *$P < 0.05$; one-way ANOVA with the Fisher's PLSD *post hoc* test (**a**,**b**). Error bars show s.e.m.

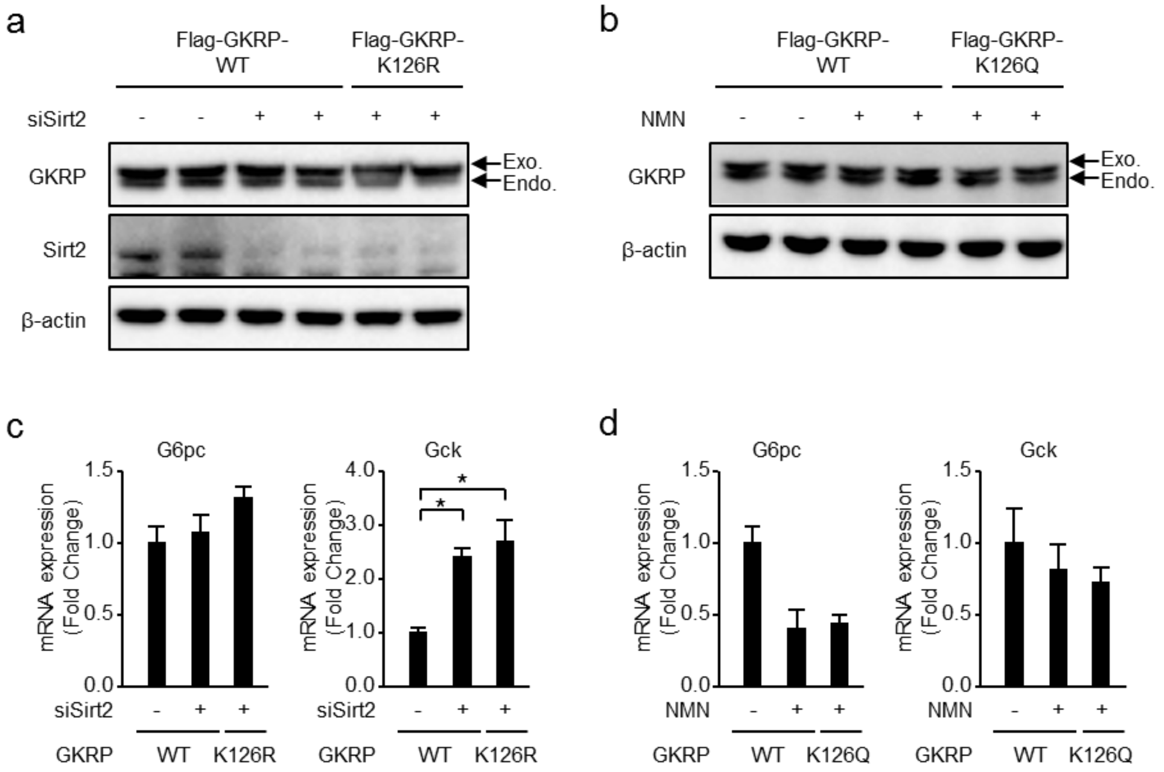

Supplementary Figure 7. GKRP-K126R or K126Q mutant has no effect on hepatic *Gck* and *G6pc* mRNA expression in mice. (**a**,**b**) Hepatic GKRP protein expression in Sirt2 knockdown mice transfected with an adenovirus for Flag-tagged GKRP- WT and Flag-tagged GKRP-K126R (**a**) and in NMN-treated HFD-fed mice transfected with an adenovirus for Flag-tagged GKRP-WT and Flag-tagged GKRP-K126Q (**b**). Endo., endogenous GKRP; Exo., exogenous GKRP. (**c**,**d**) Hepatic *G6pc* and *Gck* mRNA expression in Sirt2 knockdown mice transfected with an adenovirus for Flag-tagged GKRP- WT and Flag-tagged GKRP-K126R (**c**) and in NMN-treated HFD-fed mice transfected with an adenovirus for Flag-tagged GKRP-WT and Flag-tagged GKRP-K126Q (**d**) (n = 4). *$P < 0.05$; one-way ANOVA with the Fisher's PLSD *post hoc* test (**c**,**d**). Error bars show s.e.m.

Supplementary Figure 8

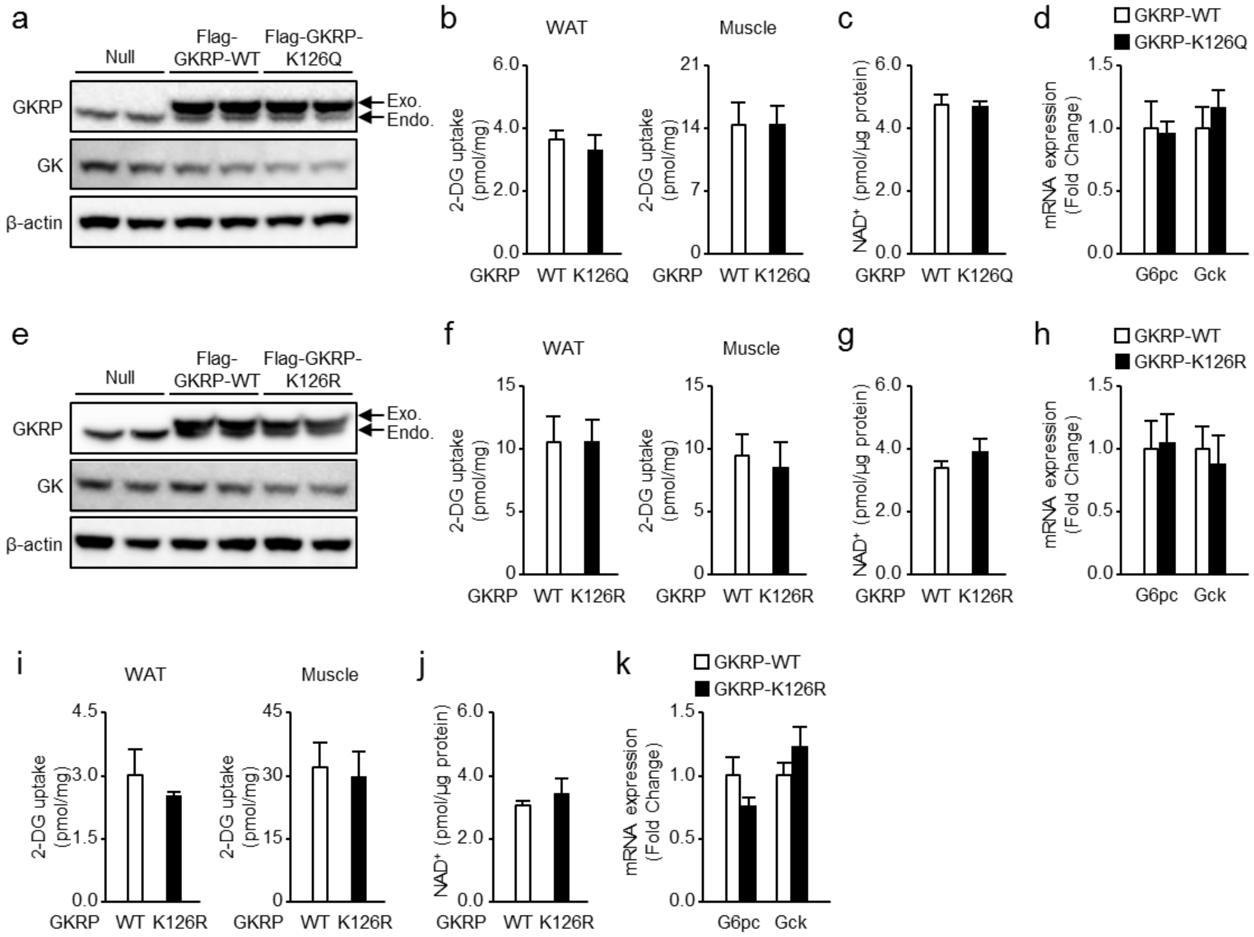

Supplementary Figure 8. GKRP-K126R or K126Q mutant has no effect on 2-DG uptake in the WAT and skeletal muscle and the gene expression of glucokinase and glucose-6-phosphatase in the liver. (**a**) Immunoblot analysis of GKRP in the liver of lean mice transfected with an adenovirus for Flag-tagged GKRP-WT or Flag-tagged GKRP-K126Q. Endo., endogenous GKRP; Exo., exogenous GKRP. (**b**–**d**) Uptake of 2-deoxyglucose (2-DG) in the white adipose tissue (WAT) and skeletal muscle (*n* = 5) (**b**), hepatic NAD+ levels (*n* = 4) (**c**) and hepatic *G6pc* and *Gck* mRNA expression (*n* = 4) (**d**) in mice with GKRP-WT or GKRP-K126Q overexpression. (**e**) Immunoblot analysis of GKRP in the liver of HFD mice transfected with an adenovirus for Flag-tagged GKRP-WT or Flag-tagged GKRP-K126R. (**f**–**h**) Uptake of 2-DG in the white adipose tissue (WAT) and skeletal muscle (*n* = 5) (**f**), hepatic NAD+ levels (*n* = 4) (**g**), and hepatic *G6pc* and *Gck* mRNA expression (*n* = 4) (**h**) in HFD mice with hepatic GKRP-WT or GKRP-K126R overexpression. (**i**–**k**) Uptake of 2-DG in the white adipose tissue (WAT) and skeletal muscle (*n* = 5) (**i**), hepatic NAD+ levels (*n* = 4) (**j**) and hepatic *G6pc* and *Gck* mRNA expression (*n* = 4) (**k**) in db/db mice with hepatic overexpression of Flag-tagged GKRP-WT or Flag-tagged GKRP-K126R. Error bars show s.e.m.

Supplementary Figure 9

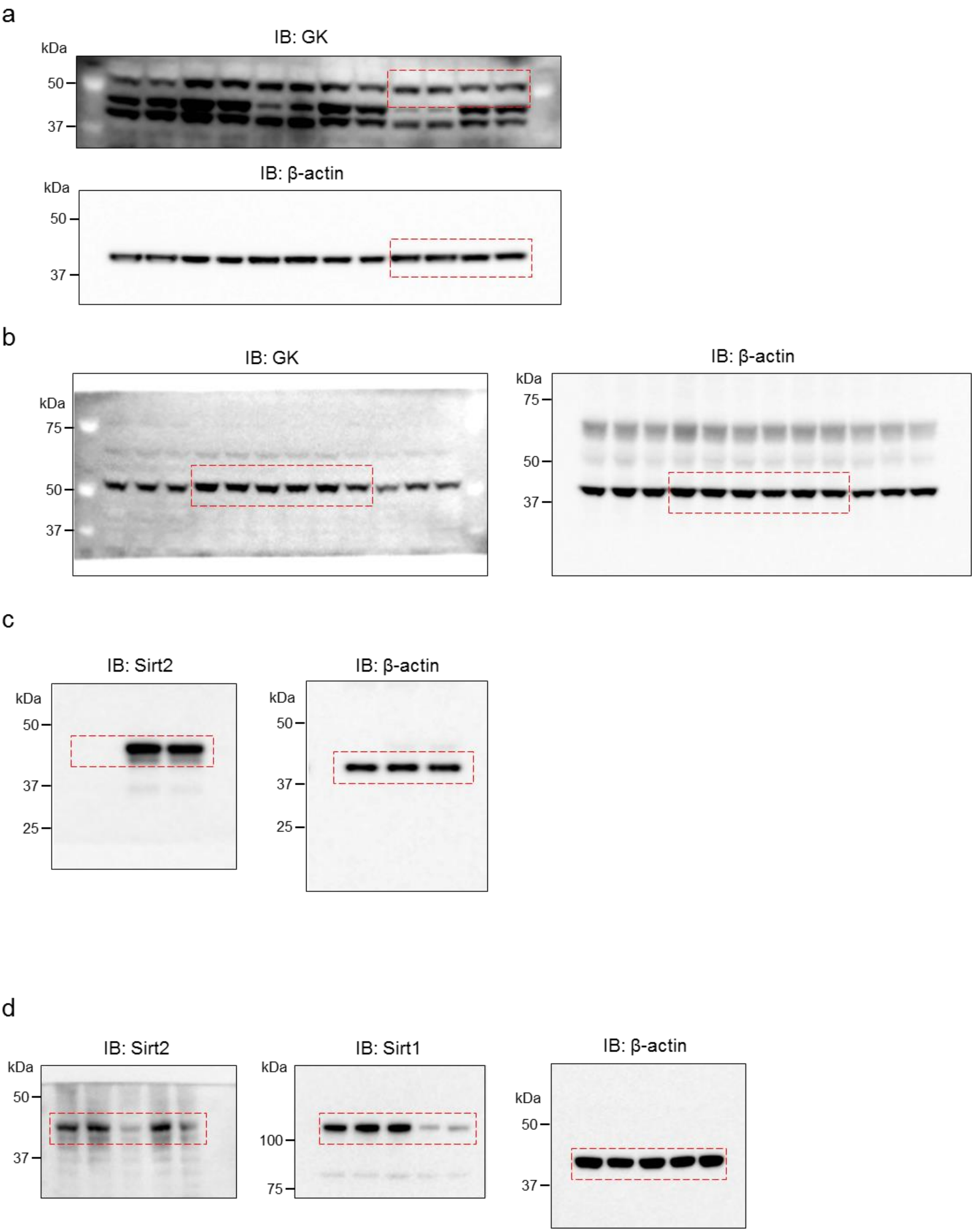

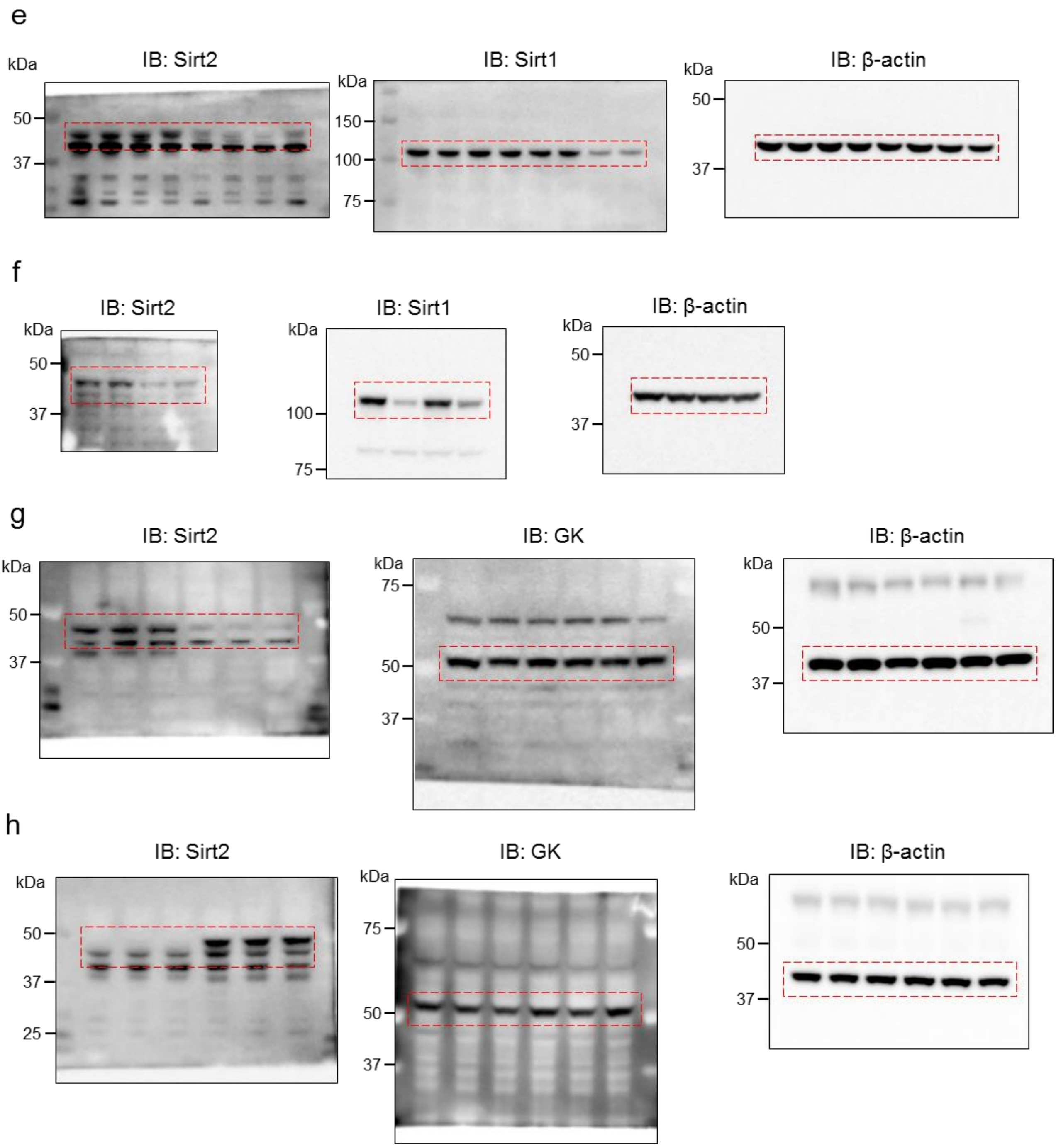

e
IB: Sirt2
kDa
50
37
IB: Sirt1
kDa
150
100
75
IB: β-actin
kDa
50
37
f
IB: Sirt2
kDa
50
37
IB: Sirt1
kDa
100
75
IB: β-actin
kDa
50
37
g
IB: Sirt2
kDa
50
37
IB: GK
kDa
75
50
37
IB: β-actin
kDa
50
37
h
IB: Sirt2
kDa
50
37
25
IB: GK
kDa
75
50
37
IB: β-actin
kDa
50
37

i

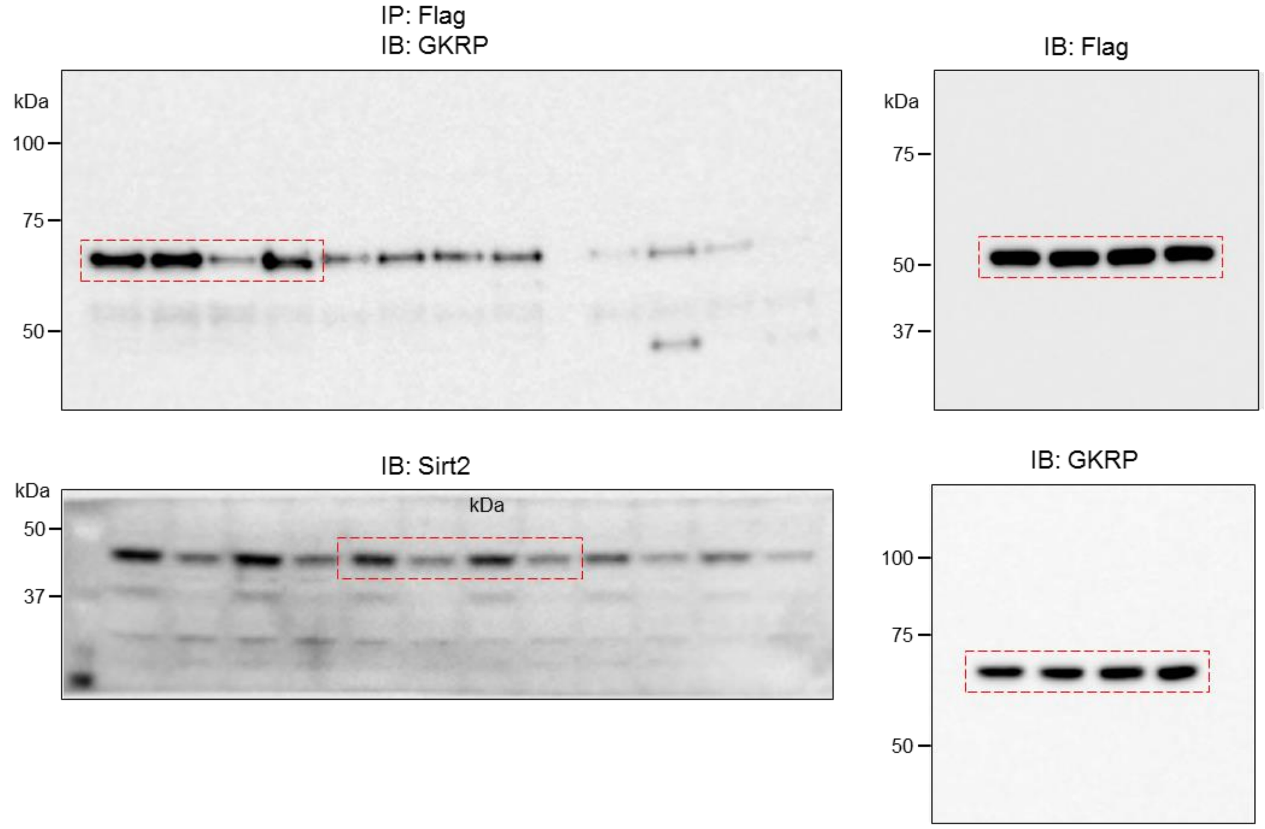
IP: Flag
IB: GKRP
kDa
100
75
50
IB: Flag
kDa
75
50
37
IB: Sirt2
kDa
50
37
kDa
IB: GKRP
100
75
50

j

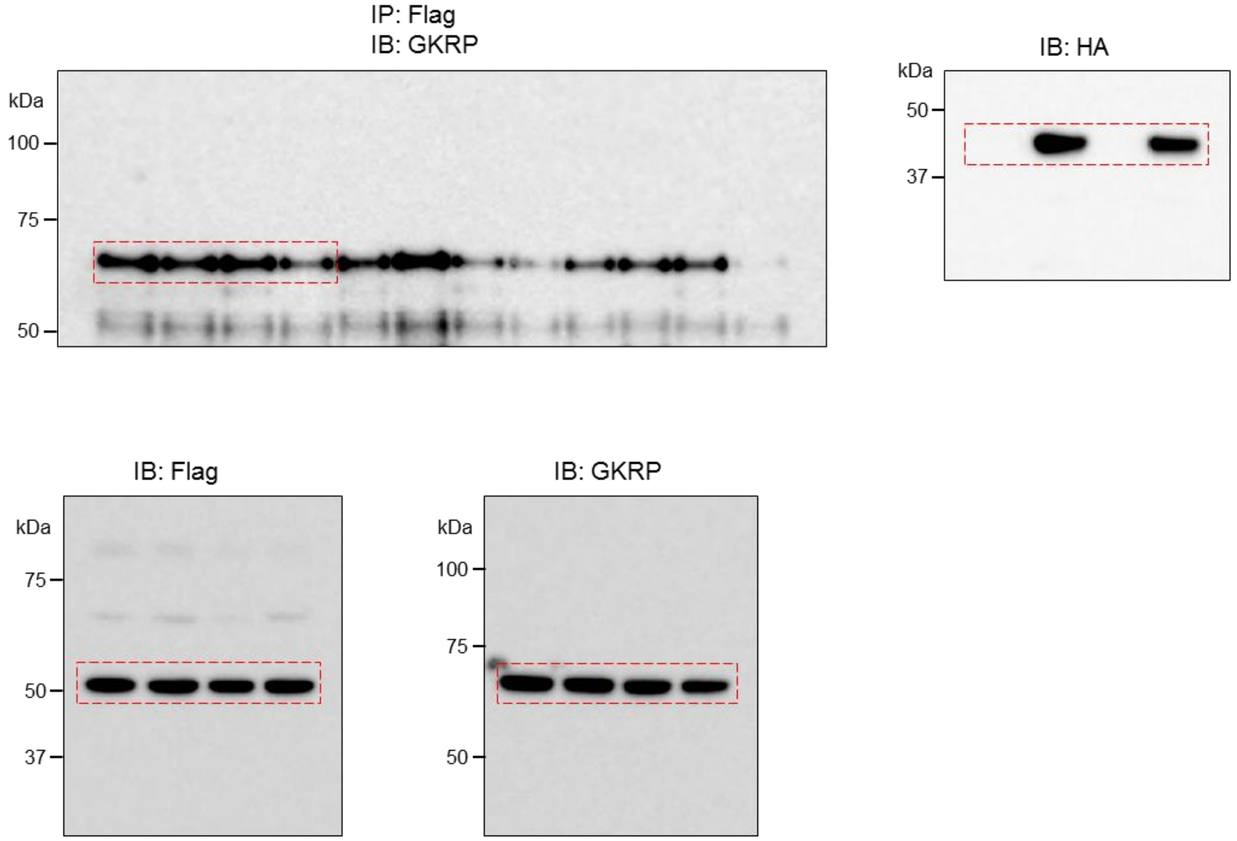
IP: Flag
IB: GKRP
kDa
100
75
50
IB: HA
kDa
50
37
IB: Flag
kDa
75
50
37
IB: GKRP
kDa
100
75
50

k

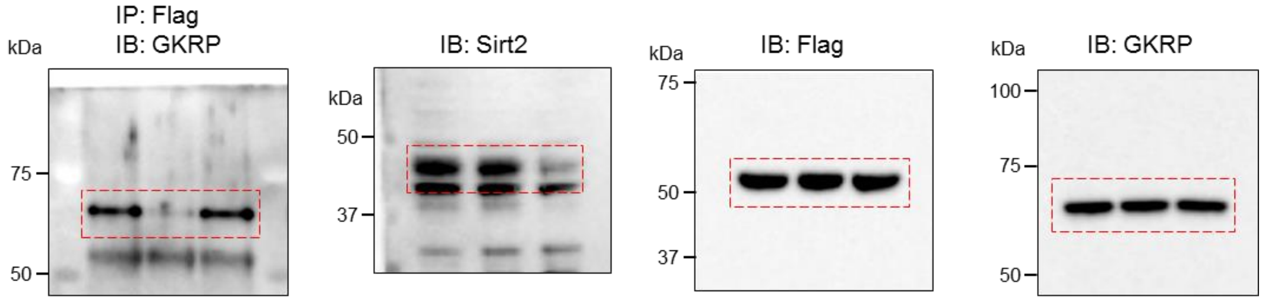
IP: Flag
IB: GKRP
kDa
75
50
IB: Sirt2
kDa
50
37
IB: Flag
kDa
75
50
37
IB: GKRP
kDa
100
75
50

l

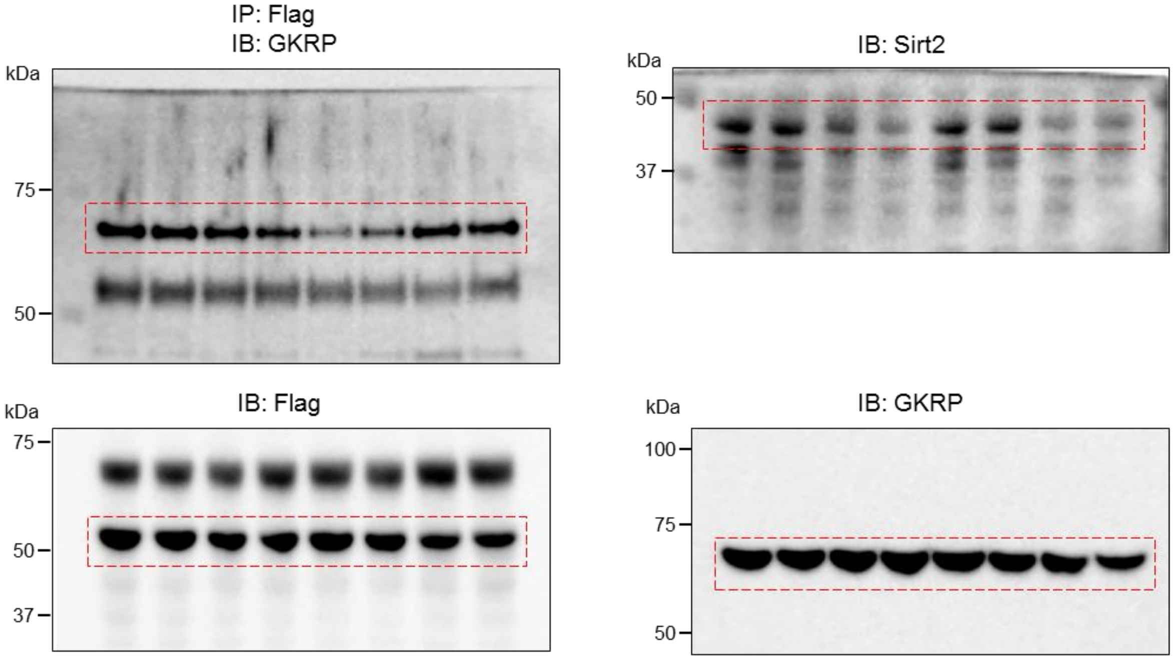
IP: Flag
IB: GKRP
kDa
75
50
IB: Sirt2
kDa
50
37
IB: Flag
kDa
75
50
37
IB: GKRP
kDa
100
75
50

m

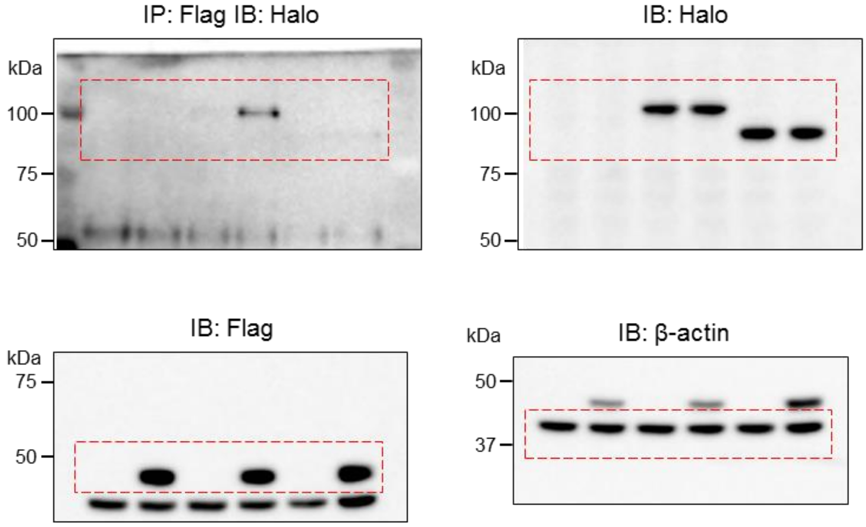
IP: Flag IB: Halo
kDa
100
75
50
IB: Halo
kDa
100
75
50
IB: Flag
kDa
75
50
IB: β-actin
kDa
50
37

n

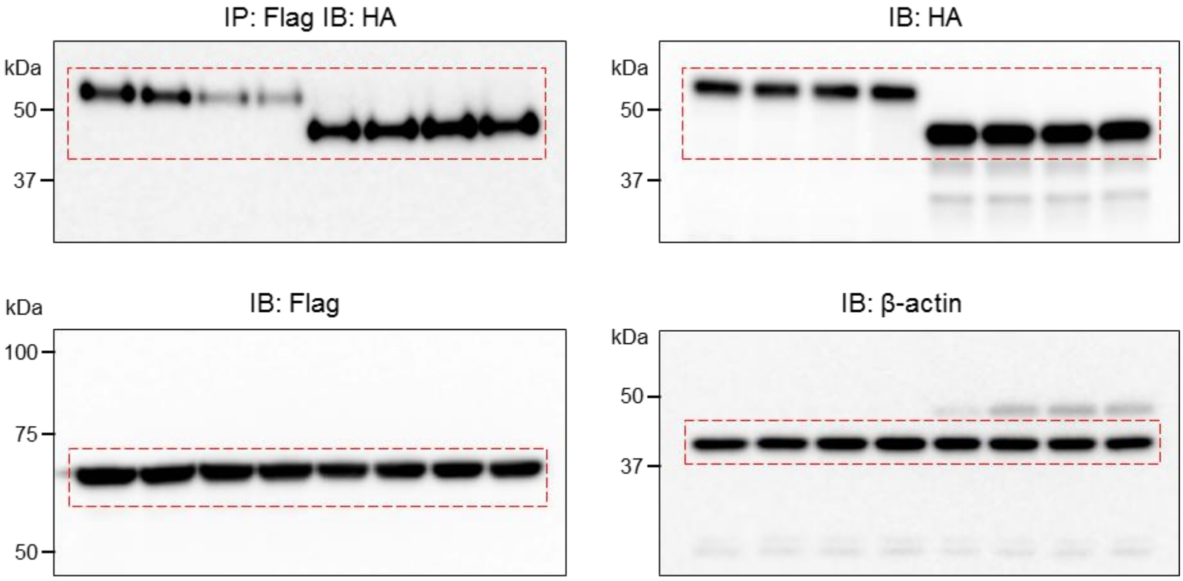

IP: Flag IB: HA
IB: HA
IB: Flag
IB: β-actin
kDa
100
75
50
37

o

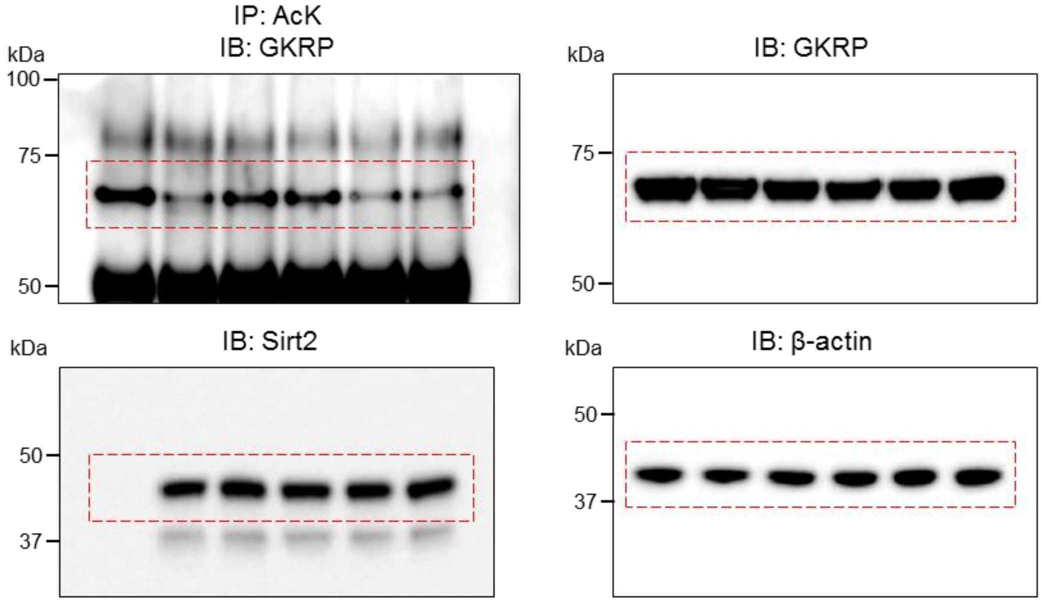

IP: AcK
IB: GKRP
IB: GKRP
IB: Sirt2
IB: β-actin
kDa
100
75
50
37

p

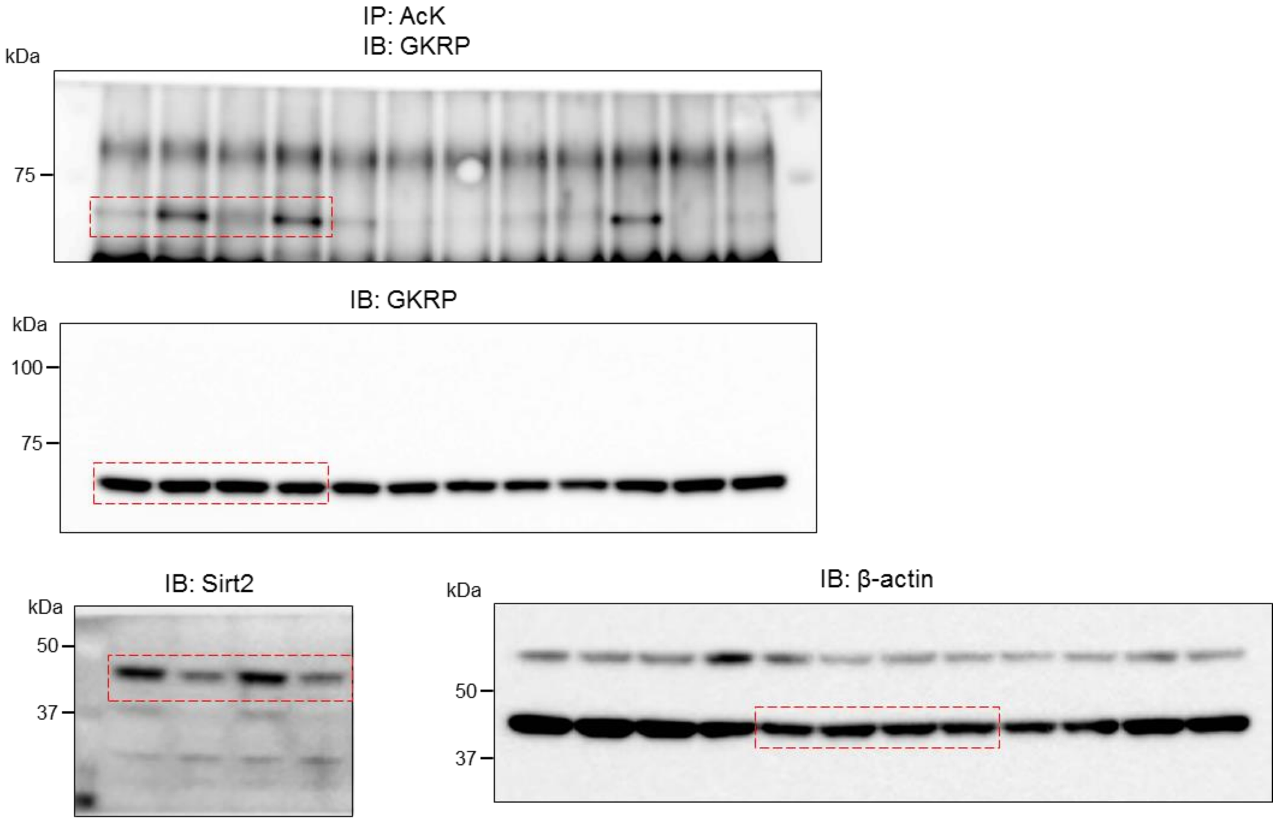
IP: AcK
IB: GKRP
kDa
75
IB: GKRP
kDa
100
75
IB: Sirt2
kDa
50
37
IB: β-actin
kDa
50
37

q

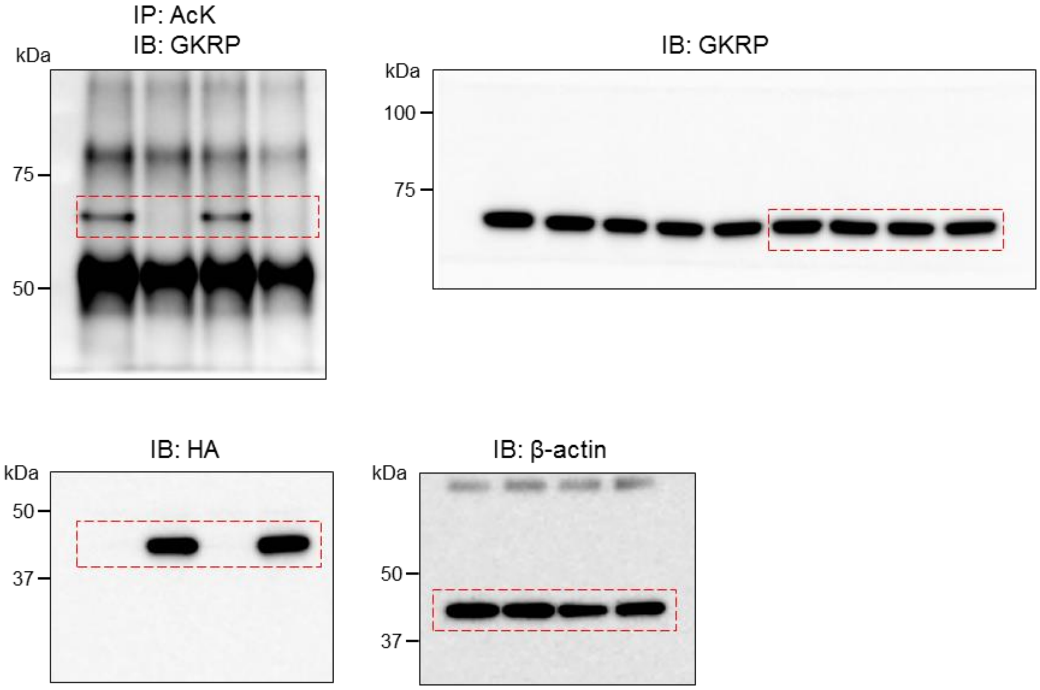
IP: AcK
IB: GKRP
kDa
75
50
IB: GKRP
kDa
100
75
IB: HA
kDa
50
37
IB: β-actin
kDa
50
37

r

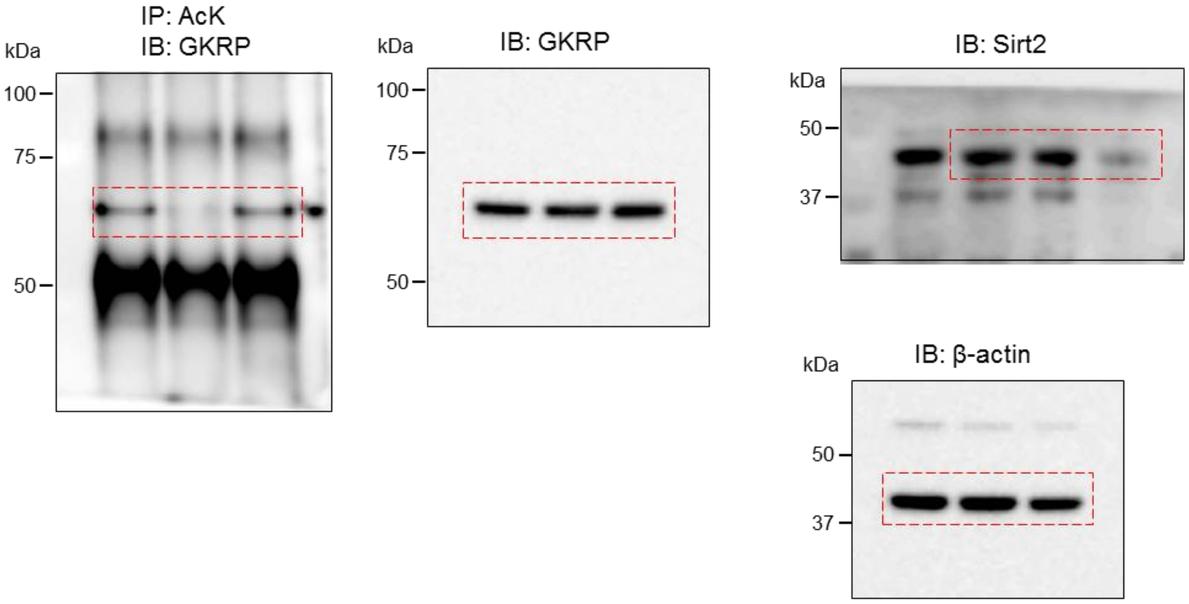
IP: AcK
IB: GKRP
kDa
100
75
50
kDa
IB: GKRP
100
75
50
kDa
IB: Sirt2
50
37
kDa
IB: β-actin
50
37

s

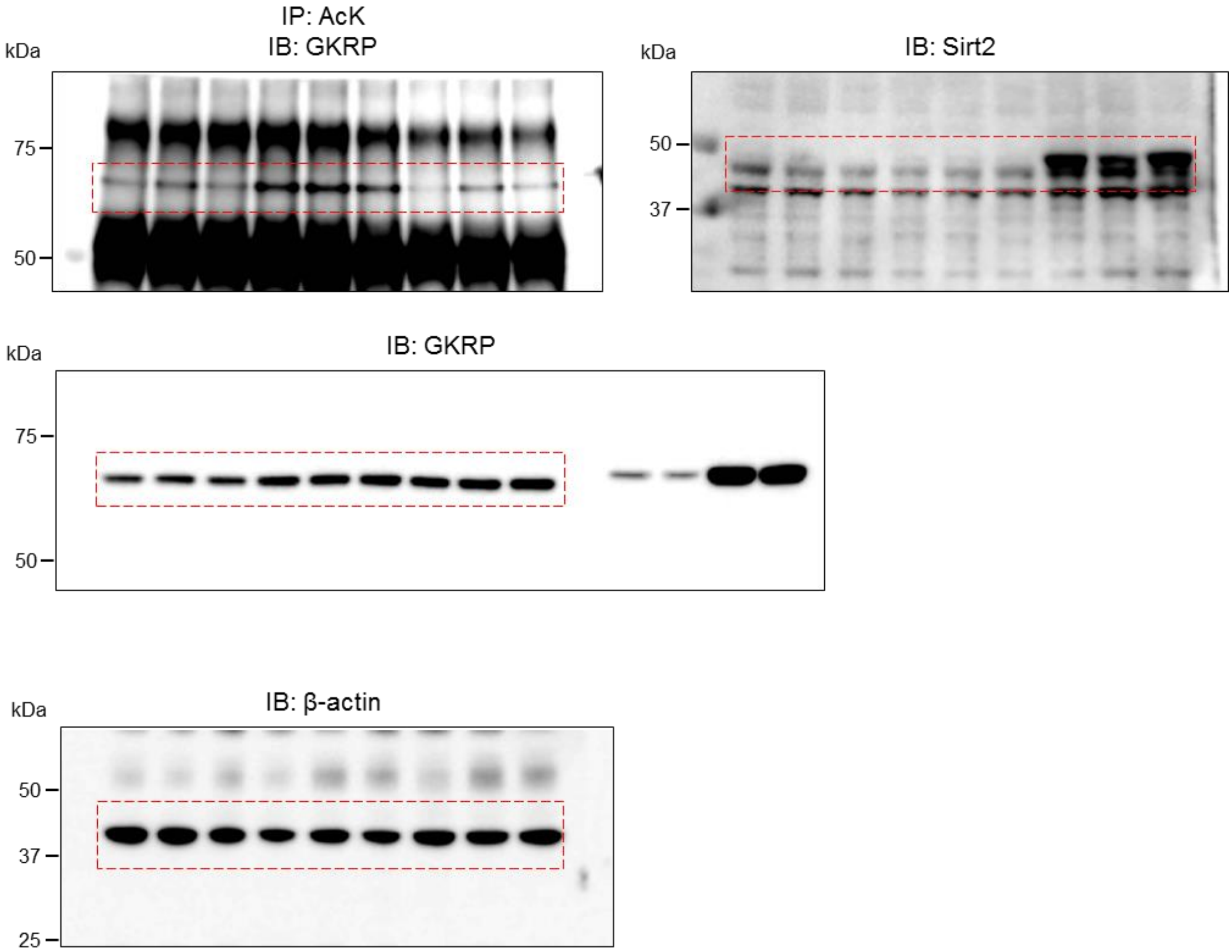
IP: AcK
IB: GKRP
kDa
75
50
kDa
IB: Sirt2
50
37
kDa
IB: GKRP
75
50
kDa
IB: β-actin
50
37
25

t

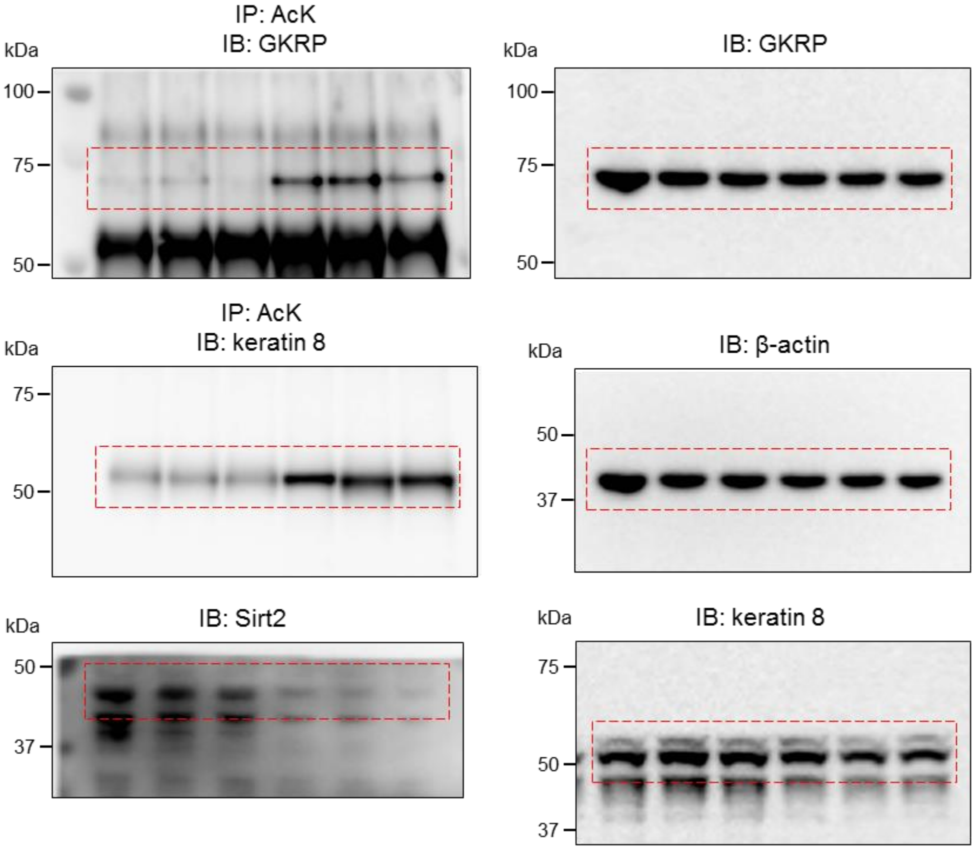
IP: AcK
IB: GKRP
kDa
100
75
50
IB: GKRP
kDa
100
75
50
IP: AcK
IB: keratin 8
kDa
75
50
IB: β-actin
kDa
50
37
IB: Sirt2
kDa
50
37
IB: keratin 8
kDa
75
50
37

u

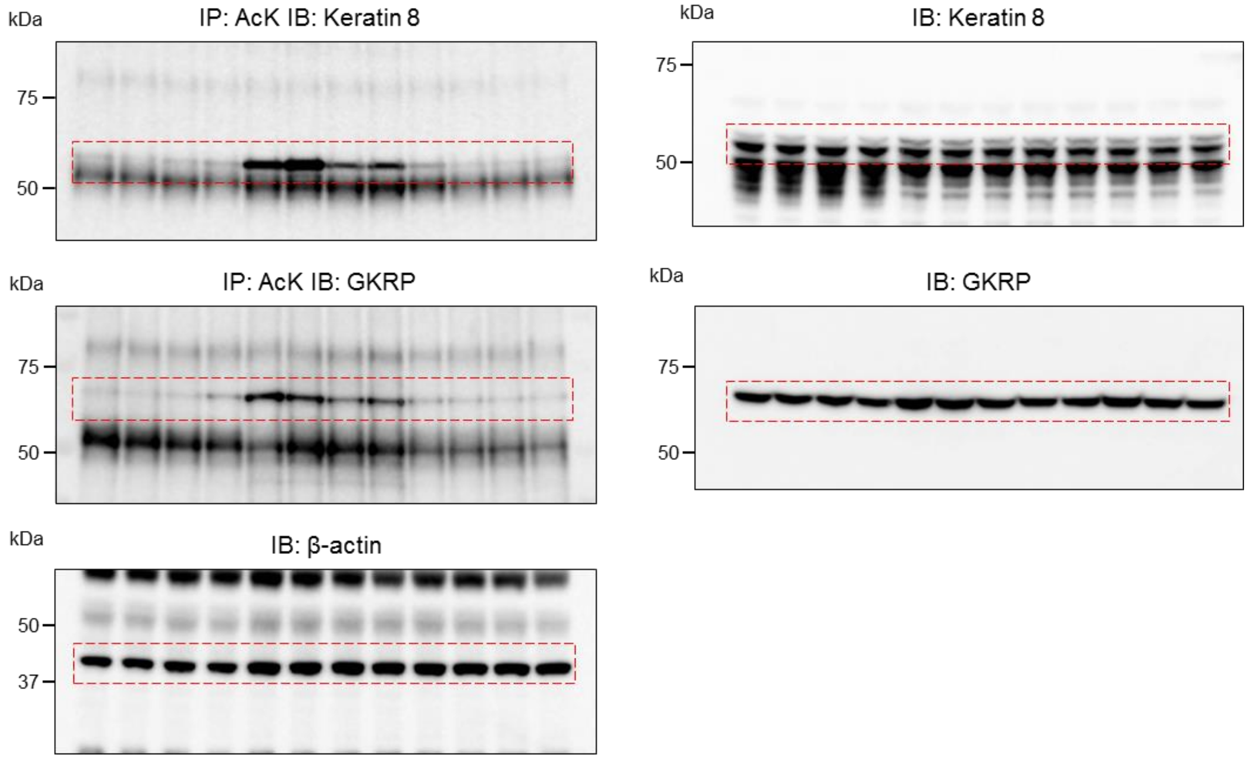
IP: AcK IB: Keratin 8
kDa
75
50
IB: Keratin 8
kDa
75
50
IP: AcK IB: GKRP
kDa
75
50
IB: GKRP
kDa
75
50
IB: β-actin
kDa
50
37

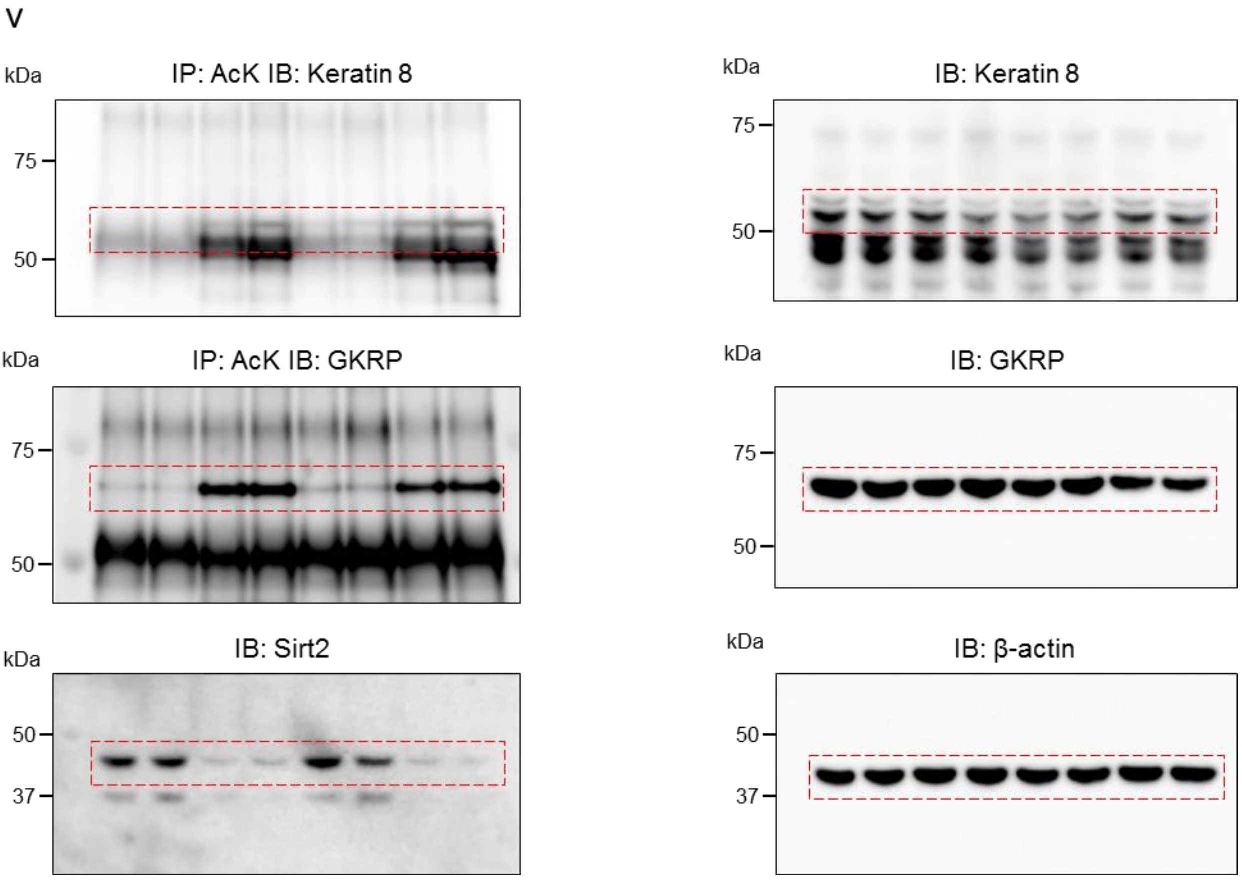

V
kDa
IP: AcK IB: Keratin 8
75
50
kDa
IB: Keratin 8
75
50
kDa
IP: AcK IB: GKRP
75
50
kDa
IB: GKRP
75
50
kDa
IB: Sirt2
50
37
kDa
IB: β-actin
50
37

W

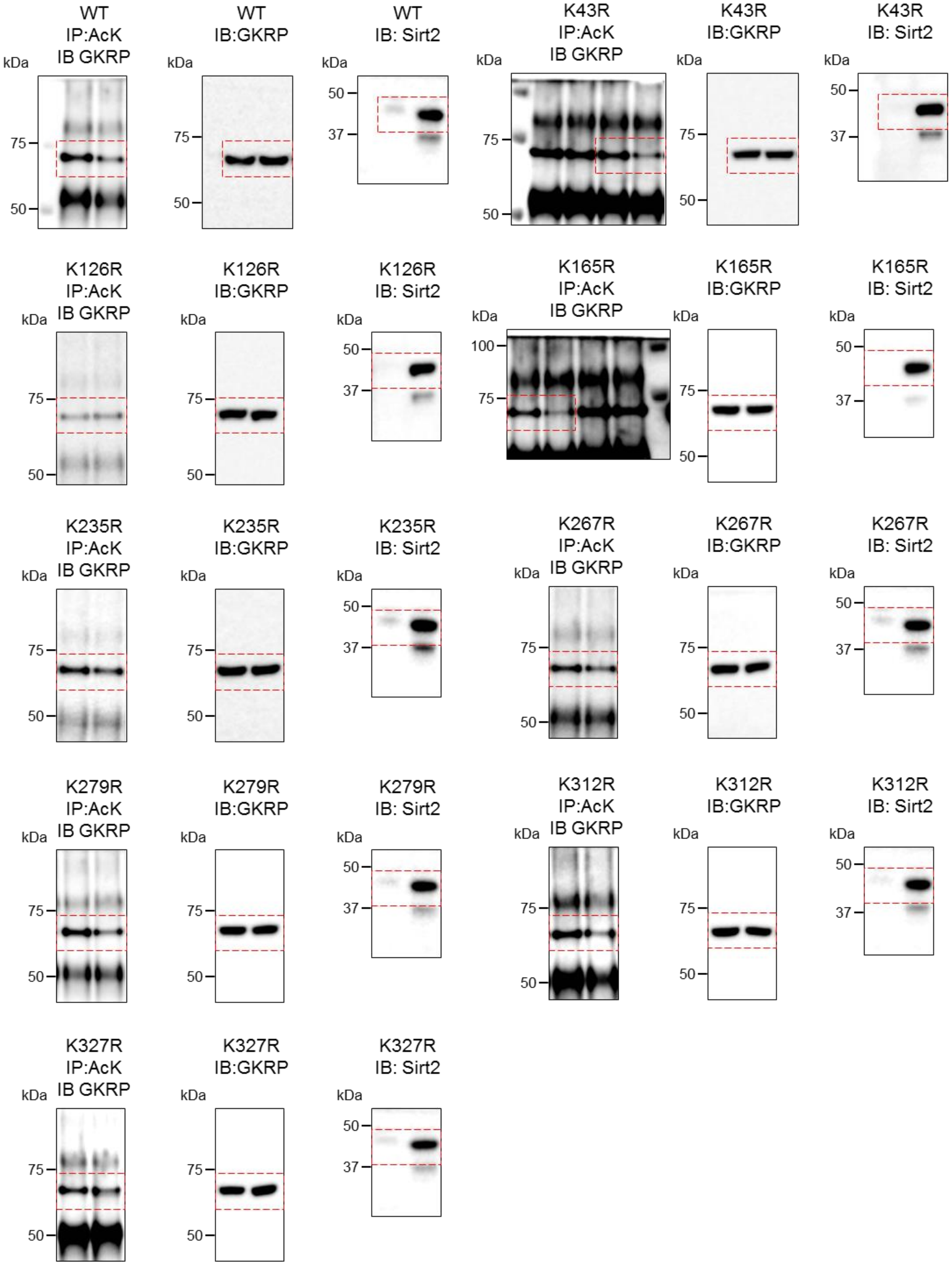
WT
IP:AcK
IB GKRP
WT
IB:GKRP
WT
IB: Sirt2
K43R
IP:AcK
IB GKRP
K43R
IB:GKRP
K43R
IB: Sirt2
K126R
IP:AcK
IB GKRP
K126R
IB:GKRP
K126R
IB: Sirt2
K165R
IP:AcK
IB GKRP
K165R
IB:GKRP
K165R
IB: Sirt2
K235R
IP:AcK
IB GKRP
K235R
IB:GKRP
K235R
IB: Sirt2
K267R
IP:AcK
IB GKRP
K267R
IB:GKRP
K267R
IB: Sirt2
K279R
IP:AcK
IB GKRP
K279R
IB:GKRP
K279R
IB: Sirt2
K312R
IP:AcK
IB GKRP
K312R
IB:GKRP
K312R
IB: Sirt2
K327R
IP:AcK
IB GKRP
K327R
IB:GKRP
K327R
IB: Sirt2
kDa
100
75
50
37

x

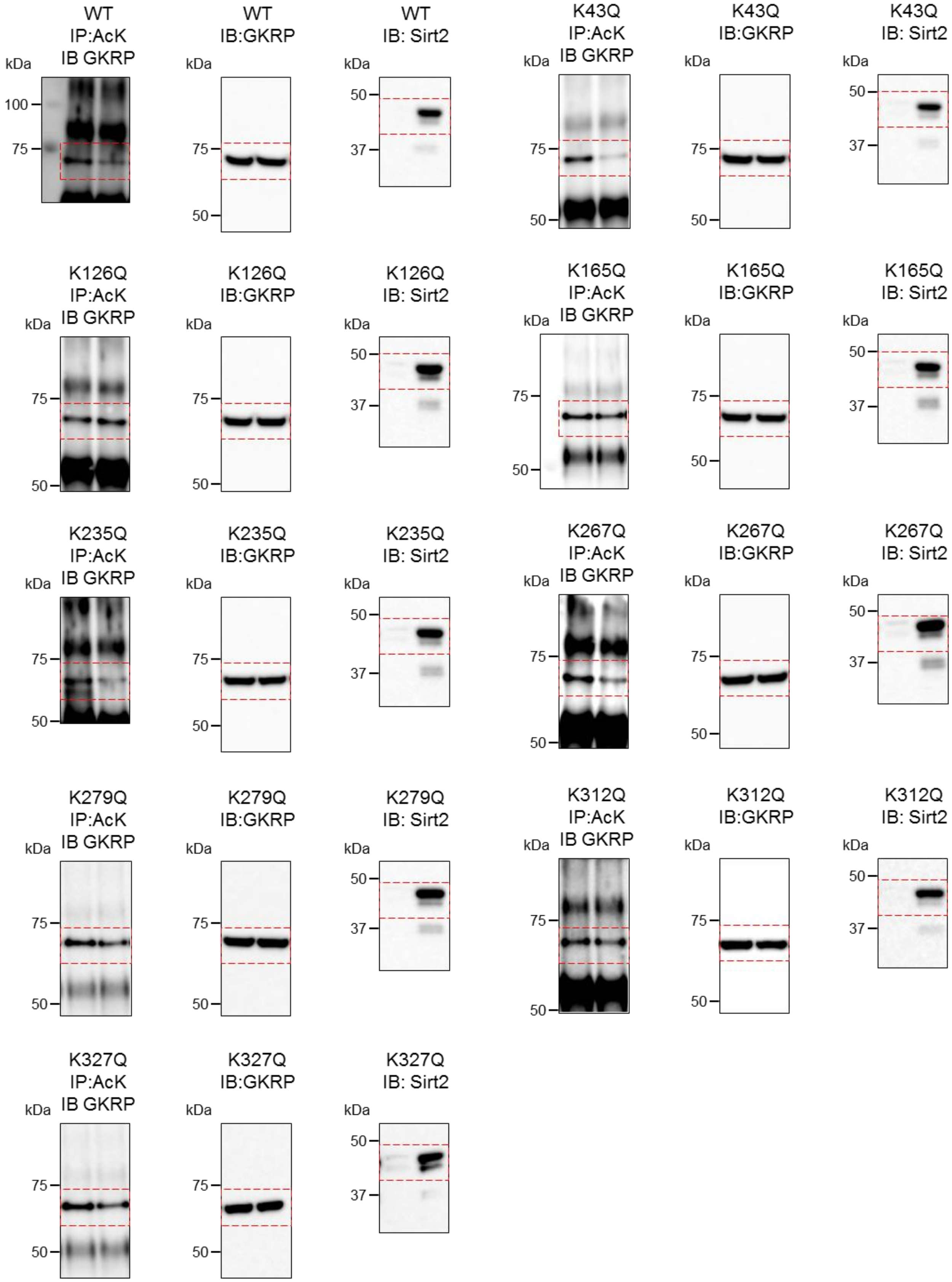

WT
IP:AcK
IB GKRP
kDa
100
75
WT
IB:GKRP
kDa
75
50
WT
IB: Sirt2
kDa
50
37
K43Q
IP:AcK
IB GKRP
kDa
75
50
K43Q
IB:GKRP
kDa
75
50
K43Q
IB: Sirt2
kDa
50
37
K126Q
IP:AcK
IB GKRP
kDa
75
50
K126Q
IB:GKRP
kDa
75
50
K126Q
IB: Sirt2
kDa
50
37
K165Q
IP:AcK
IB GKRP
kDa
75
50
K165Q
IB:GKRP
kDa
75
50
K165Q
IB: Sirt2
kDa
50
37
K235Q
IP:AcK
IB GKRP
kDa
75
50
K235Q
IB:GKRP
kDa
75
50
K235Q
IB: Sirt2
kDa
50
37
K267Q
IP:AcK
IB GKRP
kDa
75
50
K267Q
IB:GKRP
kDa
75
50
K267Q
IB: Sirt2
kDa
50
37
K279Q
IP:AcK
IB GKRP
kDa
75
50
K279Q
IB:GKRP
kDa
75
50
K279Q
IB: Sirt2
kDa
50
37
K312Q
IP:AcK
IB GKRP
kDa
75
50
K312Q
IB:GKRP
kDa
75
50
K312Q
IB: Sirt2
kDa
50
37
K327Q
IP:AcK
IB GKRP
kDa
75
50
K327Q
IB:GKRP
kDa
75
50
K327Q
IB: Sirt2
kDa
50
37

y

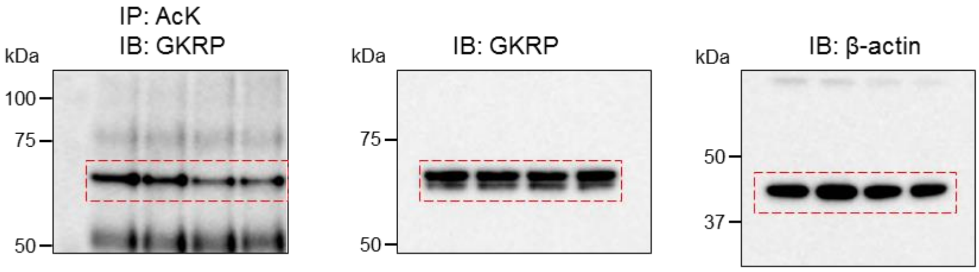
IP: AcK
IB: GKRP
kDa
100
75
50
IB: GKRP
kDa
75
50
IB: β-actin
kDa
50
37

z

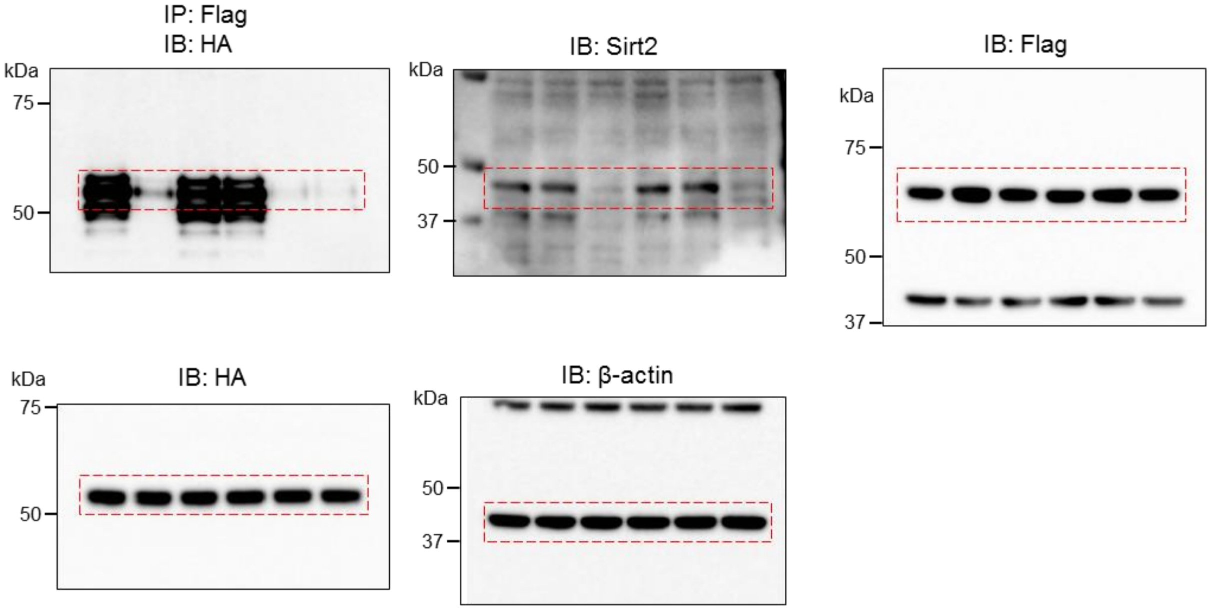
IP: Flag
IB: HA
kDa
75
50
IB: Sirt2
kDa
50
37
IB: Flag
kDa
75
50
37
IB: HA
kDa
75
50
IB: β-actin
kDa
50
37

aa

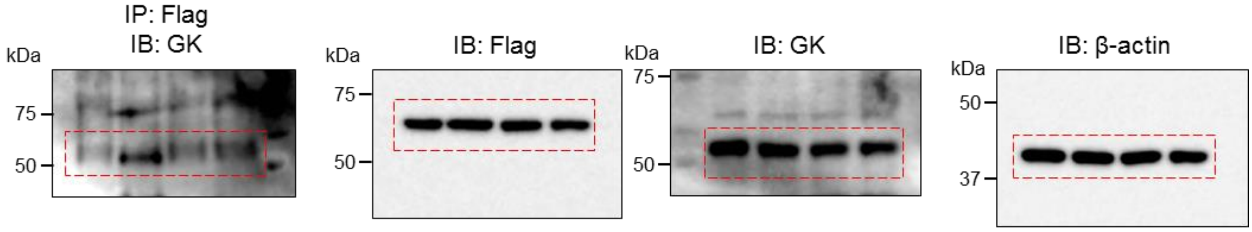
IP: Flag
IB: GK
kDa
75
50
IB: Flag
kDa
75
50
IB: GK
kDa
75
50
IB: β-actin
kDa
50
37

ab

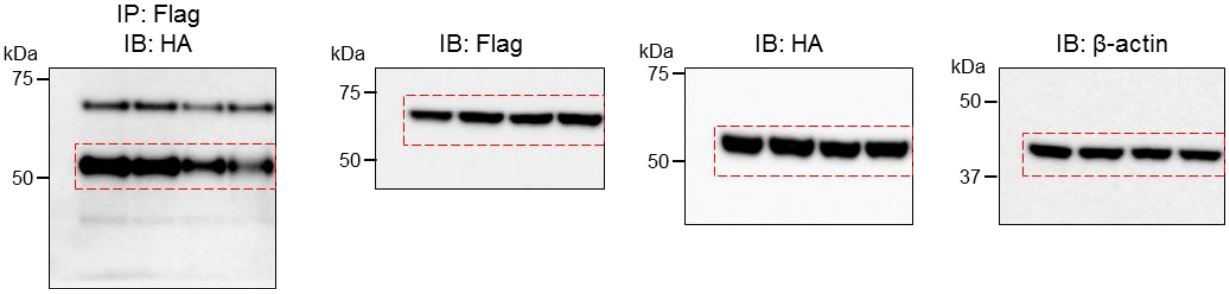
IP: Flag
IB: HA
kDa
75
50
IB: Flag
kDa
75
50
IB: HA
kDa
75
50
IB: β-actin
kDa
50
37

ac

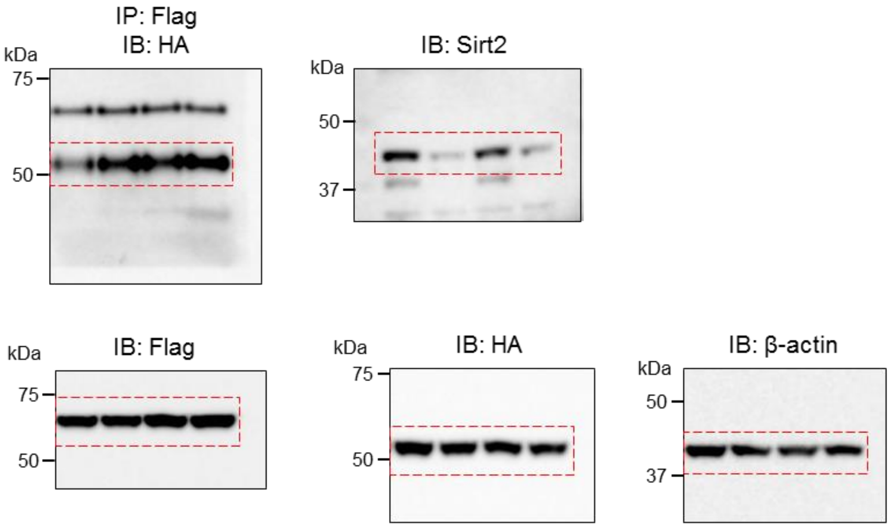
IP: Flag
IB: HA
kDa
75
50
IB: Sirt2
kDa
50
37
IB: Flag
kDa
75
50
IB: HA
kDa
75
50
IB: β-actin
kDa
50
37

ad

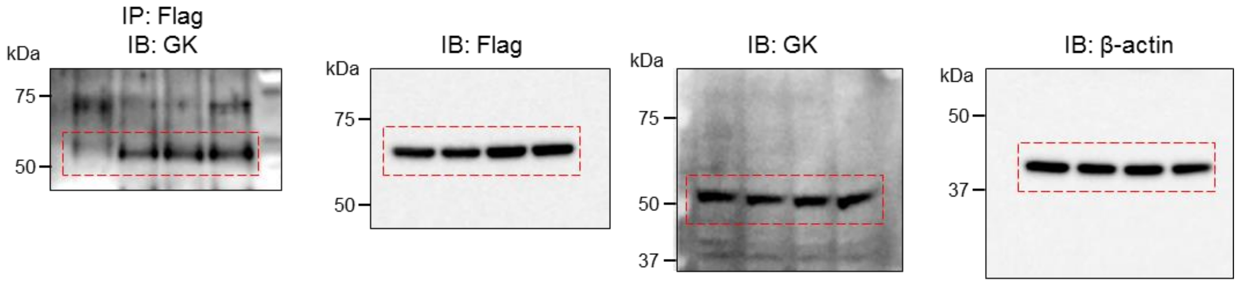
IP: Flag
IB: GK
kDa
75
50
IB: Flag
kDa
75
50
IB: GK
kDa
75
50
37
IB: β-actin
kDa
50
37

ae

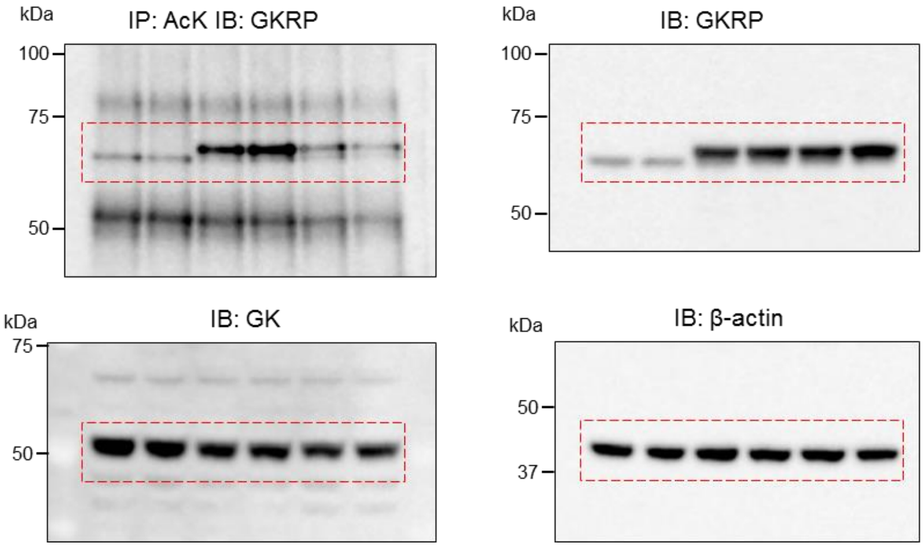
IP: AcK IB: GKRP
kDa
100
75
50
IB: GKRP
kDa
100
75
50
IB: GK
kDa
75
50
IB: β-actin
kDa
50
37

af

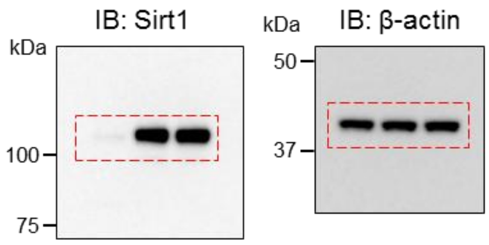
IB: Sirt1
kDa
100
75
kDa
IB: β-actin
50
37

ag

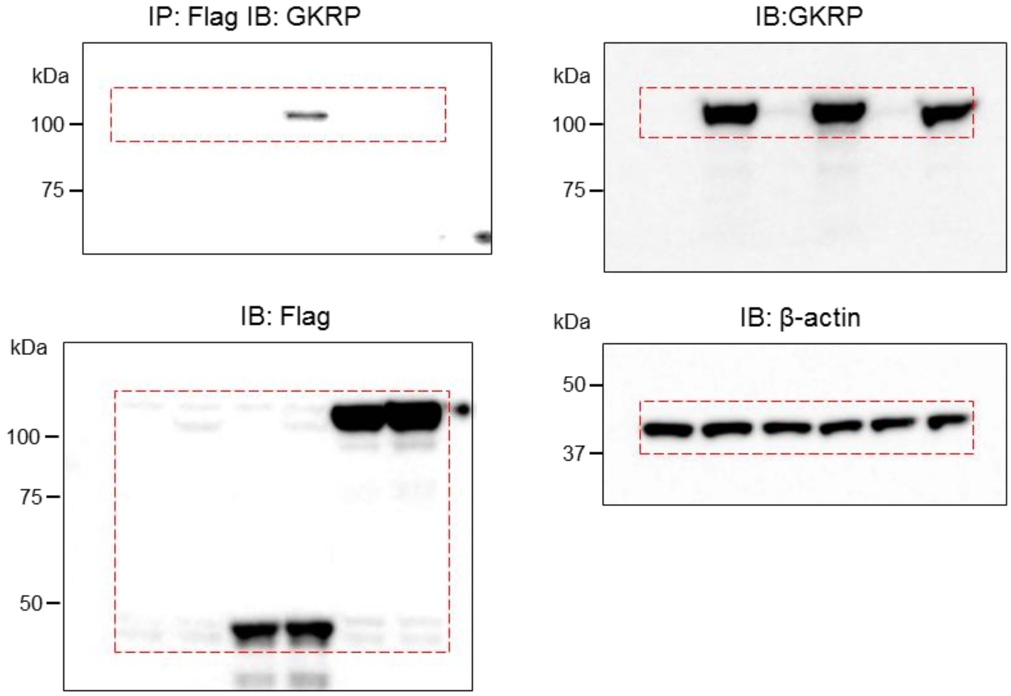
IP: Flag IB: GKRP
kDa
100
75
IB:GKRP
kDa
100
75
IB: Flag
kDa
100
75
50
kDa
IB: β-actin
50
37

ah

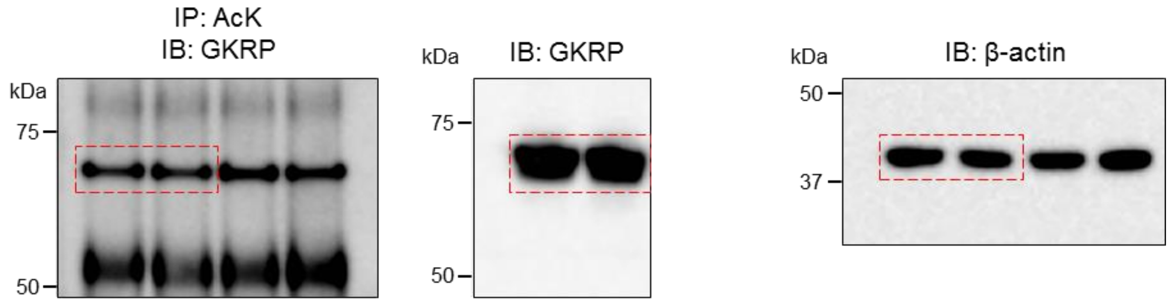
IP: AcK
IB: GKRP
kDa
75
50
kDa
IB: GKRP
75
50
kDa
IB: β-actin
50
37

ai

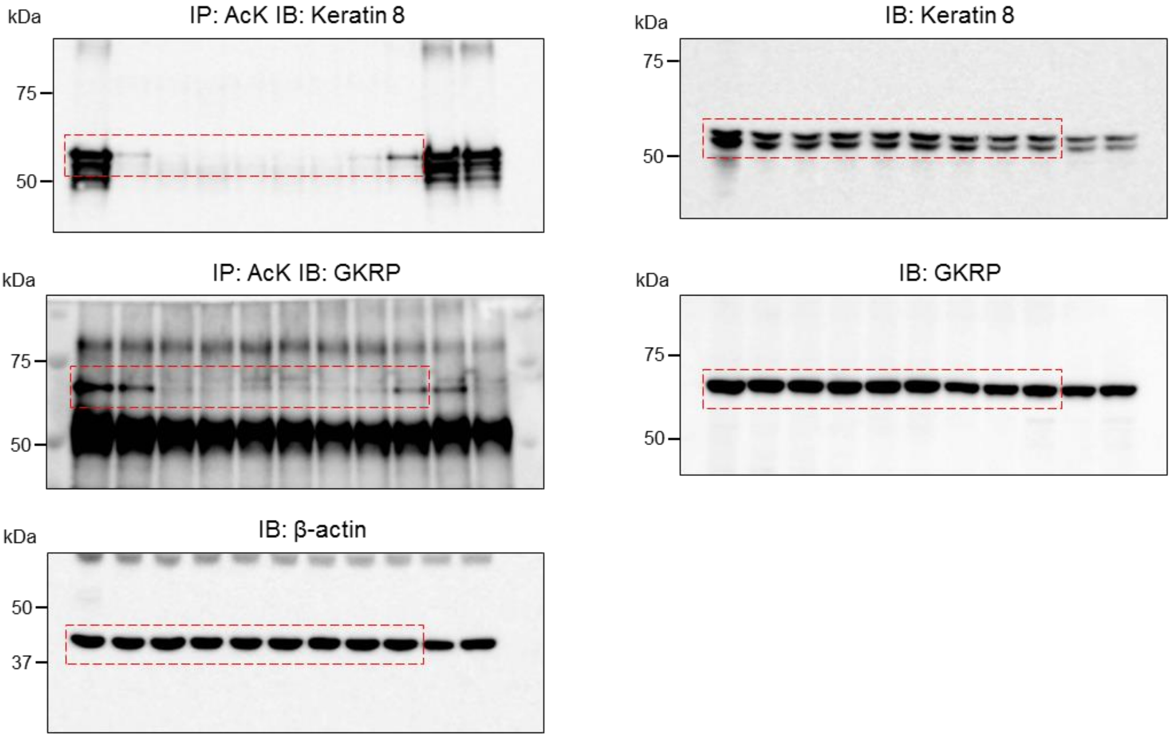
IP: AcK IB: Keratin 8
kDa
75
50
IB: Keratin 8
kDa
75
50
IP: AcK IB: GKRP
kDa
75
50
IB: GKRP
kDa
75
50
IB: β-actin
kDa
50
37

aj

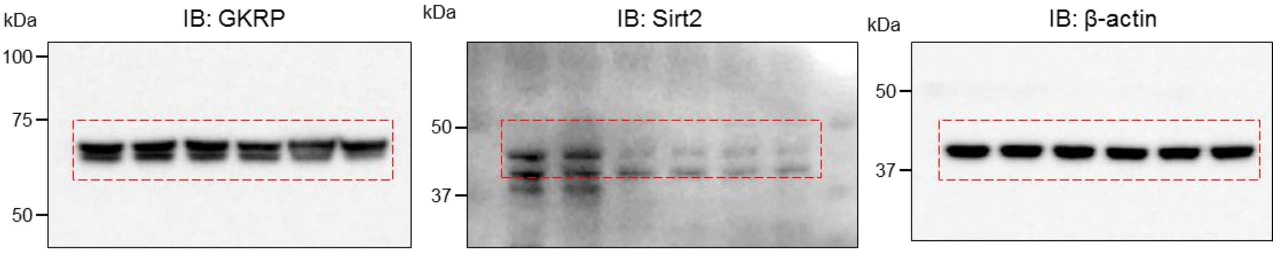
IB: GKRP
kDa
100
75
50
IB: Sirt2
kDa
50
37
IB: β-actin
kDa
50
37

ak

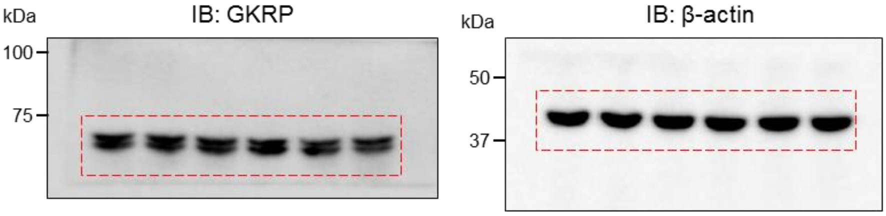
IB: GKRP
kDa
100
75
IB: β-actin
kDa
50
37

al

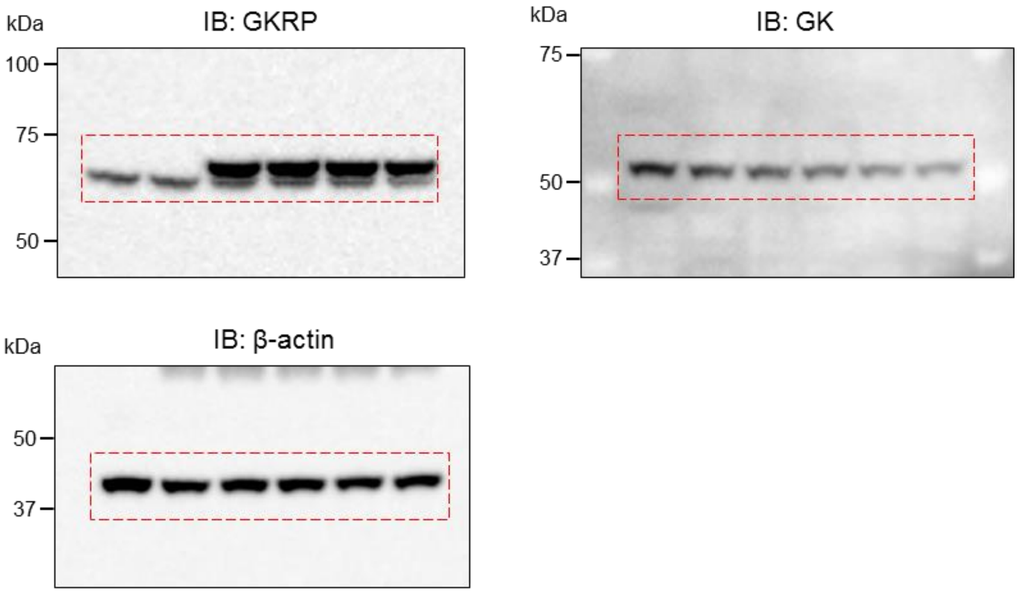

am

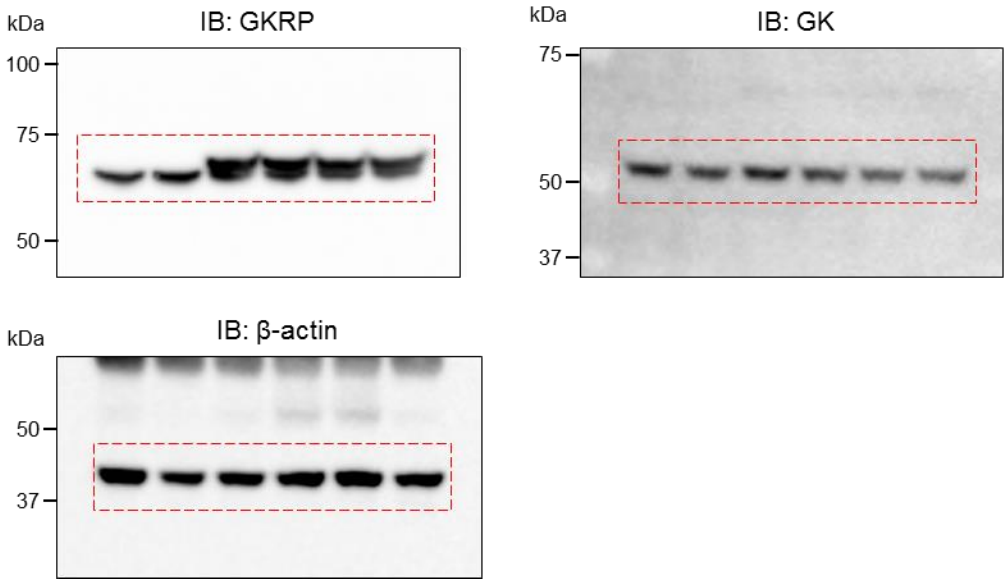

Supplementary Figure 9. Uncropped images of original scans of representative immunoblots. Uncropped versions of (**a**) Fig. 1d, (**b**) Fig. 1m, (**c**) Fig. 2c, (**d**) Fig. 2d, (**e**) Fig. 2e, (**f**) Fig. 2f, (**g**) Fig. 2g, (**h**) Fig. 2k, (**i**) Fig. 3a, (**j**) Fig. 3b, (**k**) Fig. 3c, (**l**) Fig. 3d, (**m**) Fig. 3f, (**n**) Fig. 3g, (**o**) Fig. 4a, (**p**) Fig. 4b, (**q**) Fig. 4c, (**r**) Fig. 4d, (**s**) Fig. 4e, (**t**) Fig. 4f, (**u**) Fig. 4g, (**v**) Fig. 4h, (**w**) Fig. 5a, (**x**) Fig. 5b, (**y**) Fig. 5c, (**z**) Fig. 5d, (**aa**) Fig. 5e, (**ab**) Fig. 5h, (**ac**) Fig. 5k, (**ad**) Fig. 5l, (**ae**) Fig. 7p, (**af**) Supplementary Fig. 3b, (**ag**) Supplementary Fig. 4a, (**ah**) Supplementary Fig. 4c, (**ai**) Supplementary Fig. 4d, (**aj**) Supplementary Fig. 6a, (**ak**) Supplementary Fig. 6b, (**al**) Supplementary Fig. 7a, (**am**) Supplementary Fig. 7e.
